# Exploring experiences of times without care and encounters in dementia: protocol for a living and adaptive evidence map

**DOI:** 10.1136/bmjopen-2023-075664

**Published:** 2023-09-20

**Authors:** Julian Hirt, Laura Adlbrecht, Carola Maurer, Thomas Beer

**Affiliations:** 1 Department of Health, Eastern Switzerland University of Applied Sciences, St.Gallen, Switzerland; 2 Pragmatic Evidence Lab, Research Center for Clinical Neuroimmunology and Neuroscience Basel (RC2NB), University of Basel and University Hospital Basel, Basel, Switzerland

**Keywords:** Dementia, Nursing Care, Systematic Review

## Abstract

**Introduction:**

Individuals with dementia spend most of the day without care, without encounters, and usually without activity. Although this has been proven in studies, there is a knowledge gap on how individuals with dementia experience these periods of time. Such knowledge would be highly relevant for health professionals and relatives to develop adequate strategies for dealing with these periods of time. The *FreiZeit* study aims to reconstruct periods of time without care and encounters from the perspective of individuals with dementia and formal and informal carers. The specific objective of this review is to provide a continuously updated overview of the topical evidence that may be used to guide data synthesis and interpretation within the *FreiZeit* study.

**Methods and analysis:**

We conduct a living evidence map, based on a comprehensive systematic literature search in MEDLINE/PubMed, CINAHL, PsycINFO/Ovid and Web of Science Core Collection, citation-based searches and web searches. We include studies on times without care and encounters of individuals with dementia from the perspective of individuals with dementia themselves and formal or informal caregivers of any observational study design that were conducted in the institutional and domestic long-term care setting and published as journal article in English, French or German language without any restriction of the publication year. One reviewer screens titles, abstracts and full texts and extracts data. Key characteristics and results of the included studies are charted in a tabular format. The searches will be run and continuously updated throughout the duration of the overarching *FreiZeit* study (every 6 months for 2 years from 2023 to 2025).

**Ethics and dissemination:**

Ethics approval is not required for this evidence map. We disseminate our findings via journal articles and conference proceedings as well as other formats.

**Registration details:**

This review protocol is uploaded on Open Science Framework (OSF; DOI 10.17605/OSF.IO/GDYZ9).

**Strengths and limitations of this study**

- A comprehensive literature search combining electronic key database searching, citation-based searching and web searching with no restrictions on publication date and outcomes is a methodological strength of our review.
- A living approach enables the continuous updating and monitoring of newly published studies.
- By an adaptive approach, we aim at enabling to modify the search strategy based on newly retrieved eligible studies or research questions that may be complemented.

## Introduction

For several years, the topic of dementia has gained an enormous position in the political, social and scientific debate. As the Swiss Dementia Barometer 2018 shows, the majority of the population feels well informed about dementia and believes that there are good options for supporting individuals with dementia.^1^ At the same time, however, there is a marginalisation of dementia at various levels. A review by Lara *et al* shows the connection between subjectively perceived loneliness and the risk of developing dementia.^2^ Accordingly, individuals with a subjective sense of marginalisation are particularly affected by dementia. They feel socially excluded even before diagnosis. On the other hand, the pathologising or stigmatising individuals with dementia as ‘dementia patients’ leads to an increase in marginalisation. With, despite, and because of their varying degrees of loss of cognitive and communicative abilities, they are less and less integrated into social life in the sense of social participation.

The care for individuals with dementia care is increasingly provided in a social protective environment,^3^ for example, in a family-based, dyadic care dependency or in a care facility. In Switzerland, one out of four nursing homes care for individuals with dementia in so-called protected living areas, which are mainly closed or technologies are used to surveil individuals who left the area.^4^ As a result, individuals with dementia become location-fixated, immobile and, thus, isolated.^5 6^ The lack of adequate cognitive, emotional and sensory stimulation has a negative impact on the course of dementia, the development of challenging behaviours, the shaping of the relationship work with their environment, and, above all, on the physical, cognitive, and emotional state.^7^

There is ample empirical evidence that individuals with dementia in institutional care settings experience long periods of boredom, inactivity, sleep and associated periods of loneliness,^8–11^ indicating that individuals with dementia experience large periods of time in their daily routine when they are left to their own, that is, without any encounters where they are directly addressed by another person such as caregivers, other residents, family members. However, it lacks a systematic evidence overview on the characterisation, temporal and periodical extent and how individuals with dementia and caregivers experience times without care and encounters or times in which (nearly) nothing obviously happens to and/or with individuals with dementia. Such knowledge would be highly relevant for nursing homes, health professionals and individuals’ relatives to develop adequate strategies for detecting, assessing and dealing with these periods of time.

Our primary objective is to search and map the topical evidence on times without care and encounters of people with dementia; explicitly guided by the following research questions (RQ):

- RQ1: What happens in times without care and encounters of individuals with dementia?
- RQ2: To what temporal and periodical extent do individuals with dementia experience times without care and encounter?
- RQ3: How do individuals with dementia experience times without care and encounters from their own or foreign perspective (such as caregivers and researchers)?
- RQ4: How do formal and informal caregivers experience times without care and encounters of individuals with dementia?

Our secondary objective with this review is to provide evidence for the conduct of an empirically ethnography that aims to reconstruct periods of time without care and encounters (referred to as leisure time, in German *Freizeit*) from the perspective of the individuals with dementia and from the point of view of formal or informal carers in the institutional and domestic long-term care setting in Switzerland and Germany.^12–14^ Evidence that is collected by our review may support and guide the data collection, synthesis and interpretation within the *FreiZeit* study (separate protocol in preparation^12^).

## Methods and analysis

### Design

We generate a living and adaptive evidence map, based on a comprehensive systematic literature search.^15^ An evidence map aims at mapping out, categorising and charting existing topical literature without its formal critical or quality assessment or synthesis.^16^ A living approach enables the continuous monitoring, consideration and integration of newly published studies into the body of evidence throughout the duration of the *FreiZeit* project.^12–14^ By an adaptive approach, we aim at enabling to modify the search strategy based on newly retrieved eligible studies or research questions that may be complemented. A detailed methodological outline is provided below, structured following the Preferred Reporting Items for Systematic Reviews and Meta-Analyses guidance.^17^ This review protocol is registered on Open Science Framework (OSF^18^).

### Eligibility criteria

We include studies on times without care and encounters of individuals with dementia from the perspective of individuals with dementia themselves and formal or informal caregivers of any quantitative observational and qualitative study design (ie, cohort study, cross-sectional study, survey, qualitative descriptive study, ethnography), which were conducted in the institutional and domestic long-term care setting and published as journal articles in English, French or German language without any restriction of the publication year (table 1); with no restriction to sample size and type and severity of dementia. Eligible studies refer to a sample of individuals with dementia of at least 75% of the overall study sample. A formal diagnosis of dementia is no prerequisite for individuals to be eligible for our review.

**Table 1:** Eligibility criteria following the population-interest-context (PICo) format

PICo	Inclusion criteria	Exclusion criteria
**P**opulation	Individuals with dementia * Formal caregivers † Informal caregivers ‡	Any other
Phenomenon of **I**nterest	Times without care and encounters	Other times
**C**ontext	Institutional long-term care (eg, nursing home, care home, assisted living, day care) Domestic long-term care (ie, home care)	Acute care Primary care
Design	Any type of quantitative observational or qualitative study	Evidence syntheses Experimental/interventional research
Publication type	Journal article §	Any other
Publication year	Any	–
Language	English French German	Any other

*As referred to by study authors that may mean that dementia is formally diagnosed by a physician and or assessed by health professionals such as nurses or psychologists.

†Professional caregivers such as nurses, nurse aids or activity staff.

‡Non-paid caregivers such as family members, relatives or friends.

§Irrespective of whether an article was peer-reviewed or not.

### Information sources

We search MEDLINE via PubMed, CINAHL, PsycINFO via Ovid and the Web of Science Core Collection.^19^ In addition, we conduct web searches using Google Scholar and citation searching using Scopus (ie, backward and forward citation searching of eligible publications and pertinent evidence syntheses that are retrieved during study selection until no further eligible publications can be identified)^20^ and Local Citation Network (ie, incoming and outgoing suggestions).^21^

### Search strategy

The search strategy is based on the concepts dementia (population), times without care and encounters (phenomenon of interest) and the study setting (institutional and domestic long-term care setting; table 2).

**Table 2:** Collection of search terms (as of when submitting this manuscript; without any search techniques added (eg, wildcards), without indicating controlled vocabulary)

Search component	Search terms
Dementia	ALZHEIMERS DISEASE DEMENTIA
Times without care and encounters	ASSESSMENT TOOL FOR OCCUPATION AND SOCIAL ENGAGEMENT ATOSE BORED BOREDOM BORING CARE TIME DCM DEMENTIA CARE MAPPING DEMENTIA-CARE MAPPING DISCONNECTEDNESS CONNECTEDNESS CONTACTLESS DETACHED EVERYDAY LIFE EXPERIENCE OF TIME FEELING OF TIME FREE TIME INACTIVE INACTIVITY LEISURE LIFEWORLD LONESOME MAASTRICHT ELECTRONIC DAILY LIFE OBSERVATION TOOL MEDLO-TOOL MENORAH PARK ENGAGEMENT SCALE MPES MONOTONY NO CARE NO CONTACT NO SOCIAL INTERACTION NOT ENGAGED NOT INVOLVED RESPITE RESTRAINED RETREATED SENSE OF TIME SOCIABILITY TEMPORAL PERCEPTION TIME PERCEPTION UNENGAGED UNOCCUPIED UNSTRUCTURED WITHDRAWAL WITHDRAWN
Setting (institutional and domestic long-term care)	*(institutional long-term care*) ASSISTED LIVING FACILITY CARE CENTRE CARE CENTER CARE HOME DAY CARE DAY-CARE DAY HOSPITAL DAY-HOSPITAL HOME FOR AGED HOME FOR THE AGED HOUSING FOR THE ELDERLY LONG TERM CARE LONGTERM CARE LONG-TERM CARE LONG-TERM RESIDENT LONG-TERM RESIDENTIAL LTC NURSING HOME OLD AGE HOME OLD PEOPLE'S HOME RESIDENT RESIDENTIAL AGED CARE FACILITY RESIDENTIAL FACILITY RESIDENTIAL SETTING REST HOME RETIREMENT HOME SENIOR-CITIZENS HOME SENIOR CITIZENS HOME *(domestic long-term care*) AMBULATORY CARE COMMUNITY-DWELLING DOMESTIC DOMICILIARY CARE HOME HOME BASED HOME CARE HOME HEALTH HOME HEALTH CARE HOME HEALTH NURSING HOME NURSING HOME-BASED HOME-CARE LIVING IN THE COMMUNITY NON-INSTITUTIONAL CARE NON-INSTITUTIONALIZED CARE

The collection of search terms (table 2) is based on an initial search and meta-data of its results (ie, title, abstract, author keyword(s), controlled vocabulary),^22^ brainstorming for initial search terms with members of the *FreiZeit* study team (LA and CM), reviews that were previously worked on by members of the review team and the experience in designing dementia-specific search strings of the review team^23–28^ and the results of ongoing preliminary searches (meta-data, ie, title, abstract, author keyword(s), controlled vocabulary). The controlled vocabulary search is conducted within the specific database-controlled vocabulary catalogues and is supported by the use of the Yale MeSH Analyzer,^29^ PubMed PubReminer^30^ and Coremine Medical^31^ based on references retrieved by the initial and preliminary searches.^22^

A PubMed search of MEDLINE is translated to other databases using the Polyglot Search tool^32^ and manual confirmation or adaptions by one reviewer (JH). The database-specific search strategies (for the initial search in May 2023) are provided in the online supplemental appendix 1 (any updates on the database-specific search strategies will be reported on OSF).^18^

10.1136/bmjopen-2023-075664.supp1 Supplementary data

For web searching via Google Scholar, we use a phrase derived from the research questions and title of our review (ie, ‘dementia times without care and encounters’, considering the first 20 hits). For citation searching, we perform backward citation searching (reference list checking) and forward citation searching (cited-by search) using all eligible studies and pertinent evidence syntheses (that are retrieved during study selection) as seed references via Scopus^33^; we perform iterative layers until no more additional relevant reference retrieves. In addition, we use the function of incoming and outgoing suggestions of the Local Citation Network to retrieve references that were not yet identified via other sources.^21^

All searches are carried out approximately every 6 months for the duration of the *FreiZeit* project (each May and November of the years 2023 and 2024).

### Study selection and data extraction

One reviewer (JH) screens titles, abstracts and full texts for eligibility using the Rayyan web app^34^ (title/abstract level by screening all references manually, ie, without the use of integrated machine learning features) and Covidence^35^ (full-text level). Study selection is confirmed by a second reviewer (TB), if necessary.

One reviewer (JH) extracts the following data based on the electronic full-text article version(s) using Covidence^35^ :author(s) and publication year, country of conduct, study aim or research question (as expressed by authors) and which RQs are addressed, study design, population (type of participants, sample size and type and severity of dementia (as per eligibility criteria), setting (type as expressed by authors), number of study sites, data collection (methods (eg, observation, survey), assessment instrument, perspective, and, for observations, period of time (eg, x days between month y and z), time frame (eg, between x and y o’clock) and schedule (eg, any other details provided)), data analysis methods, unit of observation (ie, observational assessment or individuals with dementia) and main results. If the reported results of individual studies are not adequately usable for the purpose of our evidence map (ie, depending on the way the data are presented), we track this accordingly, but we do not request data from study authors. Data extraction is confirmed by a second reviewer (TB), if necessary. The list of variables that are extracted is not exhaustive and may be expanded, if deemed necessary by the review and/or the *FreiZeit* team.

### Critical appraisal

As this is an evidence map that aims at providing an overview of the available topical evidence without any quality assessment, we do not critically appraise the included studies.

### Data charting

Key characteristics and results of the included studies are charted in a tabular format with columns that are based on the extracted variables (see chapter study selection and data extraction).

### Data management

One reviewer (JH) is responsible for data and literature management. A central Citavi literature database contains all search hits.^36^ Newly identified search results, irrespective of data source, are deduplicated against the existing literature in the database using the Citavi deduplication function.^36^

## Ethics and dissemination

For each update of the evidence, we provide the applied methods and a tabular overview of the studies and their results on OSF.^18^ The final version of the evidence will be aimed to publish in an open access international peer-reviewed journal.

In addition, we aim at disseminating our findings in scientific and non-scientific journal articles and conference proceedings as well as formats directed to the public and decision-makers in healthcare.

## Supplementary Material

Reviewer comments

Author's manuscript
