# Supplementary material for: Exploring experiences of times without care and encounters in dementia: protocol for a living and adaptive evidence map
Source: BMJ Open. 2023 Sep 20;13(9):e075664. doi: 10.1136/bmjopen-2023-075664 (PMC10514613; doi:10.1136/bmjopen-2023-075664)
Supplement: Supplementary data [file bmjopen-2023-075664supp001.pdf]

Exploring experiences of times without care and encounters in dementia: protocol for a living and adaptive evidence map

APPENDIX

MEDLINE via PubMed

Last search: May 11, 2023

#	Search entry	Hits
1	ALZHEIMER*[TIAB] OR DEMENT*[TIAB] OR "DEMENTIA"[MESH]	316,947
2	"ASSESSMENT TOOL OCCUPATION ENGAGEMENT"[TIAB:~3] OR ATOSE[TIAB] OR BORED[TIAB] OR BOREDOM[TIAB] OR BORING[TIAB] OR "CARE TIME"[TIAB] OR DCM[TIAB] OR "DEMENTIA CARE MAPPING"[TIAB] OR "DEMENTIA-CARE MAPPING"[TIAB] OR DISCONNECTEDNESS[TIAB] OR CONNECTEDNESS[TIAB] OR CONTACTLESS[TIAB] OR DETACHED[TIAB] OR "EVERYDAY LIFE"[TIAB] OR "EXPERIENCE TIME"[TIAB:~1] OR "FEELING TIME"[TIAB:~1] OR "FREE TIME"[TIAB] OR INACTIV*[TIAB] OR LEISURE[TIAB] OR LIFEWORLD[TIAB] OR LONESOME[TIAB] OR "MAASTRICHT ELECTRONIC DAILY LIFE OBSERVATION TOOL"[TIAB] OR "MEDLO*"[TIAB] OR "MENORAH PARK ENGAGEMENT SCALE"[TIAB] OR MPES[TIAB] OR MONOTONY[TIAB] OR "NO CARE"[TIAB] OR "NO CONTACT"[TIAB] OR "NO SOCIAL INTERACTION"[TIAB] OR "NOT ENGAGED"[TIAB] OR "NOT INVOLVED"[TIAB] OR RESPITE[TIAB] OR RESTRAINED[TIAB] OR RETREATED[TIAB] OR "SENSE TIME"[TIAB:~1] OR SOCIABILITY[TIAB] OR "TEMPORAL PERCEPTION"[TIAB] OR "TIME PERCEPTION"[TIAB] OR UNENGAGED[TIAB] OR UNOCCUPIED[TIAB] OR UNSTRUCTURED[TIAB] OR WITHDRAWAL[TIAB] OR WITHDRAWN[TIAB] OR "LEISURE ACTIVITIES"[MESH] OR "TIME PERCEPTION"[MESH]	873,861
3	"ASSISTED LIVING"[TIAB] OR "CARE CENTRE*"[TIAB] OR "CARE CENTER*"[TIAB] OR "CARE HOME*"[TIAB] OR "DAY CARE"[TIAB] OR "DAY-CARE"[TIAB] OR "DAY HOSPITAL"[TIAB] OR "DAY-HOSPITAL"[TIAB] OR "HOME* FOR AGED"[TIAB] OR "HOME* FOR THE AGED"[TIAB] OR "HOUSING FOR THE ELDERLY"[TIAB] OR "LONG TERM CARE"[TIAB] OR "LONGTERM CARE"[TIAB] OR "LONG-TERM CARE"[TIAB] OR LTC[TIAB] OR "NURSING HOME*"[TIAB] OR "OLD AGE HOME*"[TIAB] OR "OLD PEOPLE'S HOME*"[TIAB] OR RESIDENT*[TIAB] OR "REST HOME*"[TIAB] OR "RETIREMENT HOME*"[TIAB] OR "SENIOR-CITIZENS HOME*"[TIAB] OR "SENIOR CITIZENS HOME*"[TIAB] OR "HOMES FOR THE AGED"[MESH] OR "HOUSING FOR THE ELDERLY"[MESH] OR "NURSING HOMES"[MESH] OR "RESIDENTIAL FACILITIES"[MESH]	346,417
4	AMBULATORY*[TIAB] OR "COMMUNITY-DWELLING"[TIAB] OR "DOMESTIC*"[TIAB] OR DOMICILIARY*[TIAB] OR HOME*[TIAB] OR "LIVING IN THE COMMUNITY"[TIAB] OR "NON-INSTITUTIONAL*"[TIAB] OR "ADULT DAY CARE CENTERS"[MESH] OR "HOME CARE SERVICES"[MESH]	865,928
5	#3 OR #4	1,135,311
6	#1 AND #2 AND #5	1,831

CINAHL

Last search: May 11, 2023

#	Search entry	Hits
1	(TI ALZHEIMER* OR AB ALZHEIMER*) OR (TI DEMENT* OR AB DEMENT*) OR (MH DEMENTIA+)	111,658
2	(TI "ASSESSMENT TOOL FOR OCCUPATION AND SOCIAL ENGAGEMENT" OR AB "ASSESSMENT TOOL FOR OCCUPATION AND SOCIAL ENGAGEMENT") OR (TI ATOSE OR AB ATOSE) OR (TI BORED OR AB BORED) OR (TI BOREDOM OR AB BOREDOM) OR (TI BORING OR AB BORING) OR (TI "CARE TIME" OR AB "CARE TIME") OR (TI DCM OR AB DCM) OR (TI "DEMENTIA CARE MAPPING" OR AB "DEMENTIA CARE MAPPING") OR (TI "DEMENTIA-CARE MAPPING" OR "DEMENTIA-CARE MAPPING") OR (TI DISCONNECTEDNESS OR AB DISCONNECTEDNESS) OR (TI CONNECTEDNESS OR AB CONNECTEDNESS) OR (TI CONTACTLESS OR AB CONTACTLESS) OR (TI DETACHED OR AB DETACHED) OR (TI "EVERYDAY LIFE" OR AB "EVERYDAY LIFE") OR (TI (EXPERIENCE W1 TIME) OR AB (EXPERIENCE W1 TIME)) OR (TI (FEELING W1 TIME) OR AB (FEELING W1 TIME)) OR (TI "FREE TIME" OR AB "FREE TIME") OR (TI INACTIV* OR AB INACTIV*) OR (TI LEISURE OR AB LEISURE) OR (TI LIFEWORLD OR AB LIFEWORLD) OR (TI LONESOME OR AB LONESOME) OR (TI "MAASTRICHT ELECTRONIC DAILY LIFE OBSERVATION TOOL" OR AB "MAASTRICHT ELECTRONIC DAILY LIFE OBSERVATION TOOL") OR (TI MEDLO* OR AB MEDLO*) OR (TI "MENORAH PARK ENGAGEMENT SCALE" OR AB "MENORAH PARK ENGAGEMENT SCALE") OR (TI "MPES OR AB "MPES) OR (TI MONOTONY OR AB MONOTONY) OR (TI RESPITE OR AB RESPITE) OR (TI RESTRAINED OR AB RESTRAINED) OR (TI RETREATED OR AB RETREATED) OR (TI (SENSE W1 TIME) OR AB (SENSE W1 TIME)) OR (TI SOCIABILITY OR AB SOCIABILITY) OR (TI "TEMPORAL PERCEPTION" OR AB "TEMPORAL PERCEPTION") OR (TI "TIME PERCEPTION" OR AB "TIME PERCEPTION") OR (TI UNENGAGED OR AB UNENGAGED) OR (TI UNOCCUPIED OR AB UNOCCUPIED) OR (TI UNSTRUCTURED OR AB UNSTRUCTURED) OR (TI WITHDRAWAL OR AB WITHDRAWAL) OR (TI WITHDRAWN OR AB WITHDRAWN) OR MH LEISURE ACTIVITIES+	156,795
3	(TI "ASSISTED LIVING" OR AB "ASSISTED LIVING") OR (TI "CARE CENTRE*" OR AB "CARE CENTRE*") OR (TI "CARE CENTER*" OR AB "CARE CENTER*") OR (TI "CARE HOME*" OR AB "CARE HOME*") OR (TI "DAY CARE" OR AB "DAY CARE") OR (TI DAY-CARE OR AB DAY-CARE) OR (TI "DAY HOSPITAL" OR AB "DAY HOSPITAL") OR (TI DAY-HOSPITAL OR AB DAY-HOSPITAL) OR (TI "HOME* FOR AGED" OR AB "HOME* FOR AGED") OR (TI "HOME* FOR THE AGED" OR AB "HOME* FOR THE AGED") OR (TI "HOUSING FOR THE ELDERLY" OR AB "HOUSING FOR THE ELDERLY") OR (TI "LONG TERM CARE" OR AB "LONG TERM CARE") OR (TI "LONGTERM CARE" OR AB "LONGTERM CARE") OR (TI "LONG-TERM CARE" OR AB "LONG-TERM CARE") OR (TI LTC OR AB LTC) OR (TI "NURSING HOME*" OR AB "NURSING HOME*") OR (TI "OLD AGE HOME*" OR AB "OLD AGE HOME*") OR (TI "OLD PEOPLE'S HOME*" OR AB "OLD PEOPLE'S HOME*") OR (TI RESIDENT* OR AB RESIDENT*) OR (TI "REST HOME*" OR AB "REST HOME*") OR (TI "RETIREMENT HOME*" OR AB "RETIREMENT HOME*") OR (TI "SENIOR-CITIZENS HOME*" OR AB "SENIOR-CITIZENS HOME*") OR (TI "SENIOR CITIZENS HOME*" OR AB "SENIOR CITIZENS HOME*") OR MH LONG TERM CARE+ OR MH NURSING HOMES+ OR MH RESIDENTIAL CARE+	164,612
4	(TI AMBULATORY* OR AB AMBULATORY*) OR (TI "COMMUNITY-DWELLING" OR AB "COMMUNITY-DWELLING") OR (TI DOMESTIC* OR AB DOMESTIC*) OR (TI DOMICILIARY* OR AB DOMICILIARY*) OR (TI HOME* OR AB HOME*) OR (TI "LIVING W2 COMMUNITY" OR AB "LIVING W2 COMMUNITY") OR (TI NON-INSTITUTIONAL* OR AB NON-INSTITUTIONAL*) OR MH AMBULATORY CARE+ OR MH DAY CARE+ OR MH HOME HEALTH CARE+	309,297
5	#3 OR #4	422,577
6	#1 AND #2 AND #5	1,355

PsycInfo via Ovid

Last search: May 11, 2023

#	Search entry	Hits
1	(ALZHEIMER* OR DEMENT*).TI,AB. OR EXP DEMENTIA/ OR EXP ALZHEIMER'S DISEASE/	119,312
2	("ASSESSMENT TOOL FOR OCCUPATION AND SOCIAL ENGAGEMENT" OR ATOSE OR BORED OR BOREDOM OR BORING OR "CARE TIME" OR DCM OR "DEMENTIA CARE MAPPING" OR "DEMENTIA-CARE MAPPING" OR DISCONNECTEDNESS OR CONNECTEDNESS OR CONTACTLESS OR DETACHED OR "EVERYDAY LIFE" OR (EXPERIENCE ADJ1 TIME) OR (FEELING ADJ1 TIME) OR "FREE TIME" OR INACTIV* OR LEISURE OR LIFEWORLD OR LONESOME OR "MAASTRICHT ELECTRONIC DAILY LIFE OBSERVATION TOOL" OR MEDLO* OR "MENORAH PARK ENGAGEMENT SCALE" OR MPES OR MONOTONY OR "NO CARE" OR "NO CONTACT" OR "NO SOCIAL INTERACTION" OR "NOT ENGAGED" OR "NOT INVOLVED" OR RESPITE OR RESTRAINED OR RETREATED OR (SENSE ADJ1 TIME) OR SOCIABILITY OR "TEMPORAL PERCEPTION" OR "TIME PERCEPTION" OR UNENGAGED OR UNOCCUPIED OR UNSTRUCTURED OR WITHDRAWAL OR WITHDRAWN).TI,AB. OR EXP TIME PERCEPTION/ OR EXP SOCIAL WITHDRAWAL/ OR EXP SOCIABILITY/ OR EXP LEISURE TIME/	144,028
3	("ASSISTED LIVING" OR "CARE CENTRE*" OR "CARE CENTER*" OR "CARE HOME*" OR "DAY CARE" OR DAY-CARE OR "DAY HOSPITAL" OR DAY-HOSPITAL OR "HOME* FOR AGED" OR "HOME* FOR THE AGED" OR "HOUSING FOR THE ELDERLY" OR "LONG TERM CARE" OR "LONGTERM CARE" OR "LONG-TERM CARE" OR LTC OR "NURSING HOME*" OR "OLD AGE HOME*" OR "OLD PEOPLE'S HOME*" OR RESIDENT* OR "REST HOME*" OR "RETIREMENT HOME*" OR "SENIOR-CITIZENS HOME*" OR "SENIOR CITIZENS HOME*").TI,AB. OR EXP LONG TERM CARE/ OR EXP NURSING HOMES/	101,907
4	(AMBULATORY* OR COMMUNITY-DWELLING OR DOMESTIC* OR DOMICILIARY* OR HOME* OR "LIVING ADJ2 COMMUNITY" OR NON-INSTITUTIONAL*).TI,AB. OR EXP ADULT DAY CARE/ OR EXP DAY CARE CENTERS/ OR EXP HOME CARE/	229,609
5	#3 OR #4	302,197
6	#1 AND #2 AND #5	849

Hirt J, *et al*. *BMJ Open* 2023; 13:e075664. doi: 10.1136/bmjopen-2023-075664

Web of Science Core Collection

Last search: May 11, 2023

#	Search entry	Hits
1	TS=(ALZHEIMER* OR DEMENT*)	397,961
2	TS=("ASSESSMENT TOOL OCCUPATION ENGAGEMENT"[TIAB:~3] OR ATOSE OR BORED OR BOREDOM OR BORING OR "CARE TIME" OR DCM OR "DEMENTIA CARE MAPPING" OR "DEMENTIA-CARE MAPPING" OR DISCONNECTEDNESS OR CONNECTEDNESS OR CONTACTLESS OR DETACHED OR "EVERYDAY LIFE" OR (EXPERIENCE NEAR/1 TIME) OR (FEELING NEAR/1 TIME) OR "FREE TIME" OR INACTIV* OR LEISURE OR LIFEWORLD OR LONESOME OR "MAASTRICHT ELECTRONIC DAILY LIFE OBSERVATION TOOL" OR "MEDLO*" OR "MENORAH PARK ENGAGEMENT SCALE" OR MPES OR MONOTONY OR "NO CARE" OR "NO CONTACT" OR "NO SOCIAL INTERACTION" OR "NOT ENGAGED" OR "NOT INVOLVED" OR RESPITE OR RESTRAINED OR RETREATED OR (SENSE NEAR/1 TIME) OR SOCIABILITY OR "TEMPORAL PERCEPTION" OR "TIME PERCEPTION" OR UNENGAGED OR UNOCCUPIED OR UNSTRUCTURED OR WITHDRAWAL OR WITHDRAWN)	1,010,148
3	TS=("ASSISTED LIVING" OR "CARE CENTRE*" OR "CARE CENTER*" OR "CARE HOME*" OR "DAY CARE" OR "DAY-CARE" OR "DAY HOSPITAL" OR "DAY-HOSPITAL" OR "HOME* FOR AGED" OR "HOME* FOR THE AGED" OR "HOUSING FOR THE ELDERLY" OR "LONG TERM CARE" OR "LONGTERM CARE" OR "LONG-TERM CARE" OR LTC OR "NURSING HOME*" OR "OLD AGE HOME*" OR "OLD PEOPLE'S HOME*" OR RESIDENT* OR "REST HOME*" OR "RETIREMENT HOME*" OR "SENIOR-CITIZENS HOME*" OR "SENIOR CITIZENS HOME*")	490,186
4	TS=(AMBULATORY* OR "COMMUNITY-DWELLING" OR "DOMESTIC*" OR DOMICILIARY* OR HOME* OR "LIVING IN THE COMMUNITY" OR "NON-INSTITUTIONAL*")	1,341,552
5	#3 OR #4	1,727,276
6	#1 AND #2 AND #5	2,071

Hirt J, *et al. BMJ Open* 2023; 13:e075664. doi: 10.1136/bmjopen-2023-075664
