# Supplementary material for: Exploring experiences of times without care and encounters in dementia: protocol for a living and adaptive evidence map
Source: BMJ Open. 2023 Sep 20;13(9):e075664. doi: 10.1136/bmjopen-2023-075664 (PMC10514613; doi:10.1136/bmjopen-2023-075664)
Supplement: Author's manuscript [file bmjopen-2023-075664.draft_revisions.pdf]

BMJ Open is committed to open peer review. As part of this commitment we make the peer review history of every article we publish publicly available.

When an article is published we post the peer reviewers' comments and the authors' responses online. We also post the versions of the paper that were used during peer review. These are the versions that the peer review comments apply to.

The versions of the paper that follow are the versions that were submitted during the peer review process. They are not the versions of record or the final published versions. They should not be cited or distributed as the published version of this manuscript.

BMJ Open is an open access journal and the full, final, typeset and author-corrected version of record of the manuscript is available on our site with no access controls, subscription charges or pay-per-view fees (http://bmjopen.bmj.com).

If you have any questions on BMJ Open's open peer review process please email info.bmjopen@bmj.com

BMJ Open

Exploring experiences of times without care and encounters in dementia: protocol for a living and adaptive evidence map

Journal:	*BMJ Open*
Manuscript ID	bmjopen-2023-075664
Article Type:	Protocol
Date Submitted by the Author:	15-May-2023

Complete List of Authors:	Hirt, Julian; Eastern Switzerland University of Applied Sciences, Department of Health; University of Basel, Department of Clinical Research<br>Adlbrecht , Laura ; Eastern Switzerland University of Applied Sciences, Department of Health<br>Maurer , Carola ; Eastern Switzerland University of Applied Sciences, Department of Health<br>Beer, Thomas ; Eastern Switzerland University of Applied Sciences, Department of Health

Keywords:	Dementia, Nursing Care, Systematic Review

SCHOLARONE™ Manuscripts

Exploring experiences of times without care and encounters in dementia: protocol for a living and adaptive evidence map

Julian Hirt [1,2,3], Laura Adlbrecht [1], Carola Maurer [1], Thomas Beer [1] for the *FreiZeit* study team

Affiliation(s)
[1] Eastern Switzerland University of Applied Sciences, Department of Health, St.Gallen, Switzerland
[2] University Hospital Basel and University of Basel, Research Center for Clinical Neuroimmunology and Neuroscience Basel (RC2NB), Basel, Switzerland
[3] University Hospital Basel and University of Basel, Department of Clinical Research, Basel, Switzerland

FreiZeit study team members and their affiliations: https://projekt-freizeit.com/?page_id=70

Authors' ORCID and email
Julian Hirt: 0000-0001-6589-3936, julian.hirt@ost.ch
Laura Adlbrecht: 0000-0002-7042-7523, laura.adlbrecht@ost.ch
Carola Maurer: 0000-0002-2731-7767, carola.maurer@ost.ch
Thomas Beer: -, thomas.beer@ost.ch

Correspondence
Julian Hirt, Eastern Switzerland University of Applied Sciences, Department of Health, Rosenbergstrasse 59, St.Gallen, Switzerland, julian.hirt@ost.ch

For peer review only

ABSTRACT

Introduction

Individuals with dementia spend most of the day without care, without encounters, and usually without activity. Although this has been proven in studies, there is a knowledge gap on how individuals with dementia experience these periods of time. Such knowledge would be highly relevant for health professionals and relatives to develop adequate strategies for dealing with these periods of time. The *FreiZeit* study aims to reconstruct periods of time without care and encounters from the perspective of individuals with dementia and formal and informal carers. The specific objective of this review is to provide a continuously updated overview of the topical evidence that may be used to guide data synthesis and interpretation within the *FreiZeit* study.

Methods and analysis

We conduct a living evidence map, based on a comprehensive systematic literature search in MEDLINE via PubMed, CINAHL, PsycInfo via Ovid, and Web of Science Core Collection, citation-based searches, and web searches. We include studies on times without care and encounters of individuals with dementia from the perspective of individuals with dementia themselves and formal or informal caregivers of any design (i.e., primary studies and evidence synthesis) published as journal article in English, French, or German language without any restriction of the publication year. Two reviewers screen titles, abstracts, and full texts; one reviewer extracts data. Key characteristics and results of the included studies are charted in a tabular format. The searches will be run and continuously updated throughout the duration of the overarching *FreiZeit* project (approximately every six months for three years from 2023 to 2025).

Ethics and dissemination

Ethics approval is not required for this evidence map. We will disseminate our findings via journal articles and conference proceedings as well as other formats.

Registration details

This review protocol is uploaded on Open Science Framework (OSF; DOI 10.17605/OSF.IO/GDYZ9).

Keywords

Dementia [MeSH]; Nursing Homes [MeSH]; Home Nursing [MeSH]; Home Care Services [MeSH]; Review [MeSH]

For peer review only - http://bmjopen.bmj.com/site/about/guidelines.xhtml

Strengths and limitations of this study

- A comprehensive literature search combining electronic key database searching, citation-based searching, and web searching with no restrictions on publication date and outcomes is a methodological strength of our review.
- A living approach enables the continuous updating and monitoring of newly published studies.
- By an adaptive approach, we aim at enabling to modify the search strategy based on newly retrieved eligible studies or research questions that may be complemented.
- Since we do not expect many studies that were conducted to answer our review questions (assumption based on preliminary searches on the topic), the available evidence may be limited.

For peer review only

For peer review only - http://bmjopen.bmj.com/site/about/guidelines.xhtml

INTRODUCTION

For several years, the topic of dementia has gained an enormous position in the political, social, and scientific debate. As the Swiss Dementia Barometer 2018 shows, the majority of the population feels well informed about dementia and believes that there are good options for supporting individuals with dementia [1]. At the same time, however, there is a marginalization of dementia at various levels. A review by Lara et al. shows the connection between subjectively perceived loneliness and the risk of developing dementia [2]. Accordingly, individuals with a subjective sense of marginalization are particularly affected by dementia. They feel socially excluded even before diagnosis. On the other hand, the pathologizing or stigmatizing individuals with dementia as "dementia patients" leads to an increase in marginalization. With, despite, and because of their varying degrees of loss of cognitive and communicative abilities, they are less and less integrated into social life in the sense of social participation.

The care for individuals with dementia care is increasingly provided in a social protective environment [3], for example in a family-based, dyadic care dependency or in a care facility. In Switzerland, one out of four nursing homes cares for individuals with dementia in so-called protected living areas, which are mainly closed or technologies are used to surveil individuals who left the area [4]. As a result, individuals with dementia become location-fixated, immobile, and thus isolated [5, 6]. The lack of adequate cognitive, emotional, and sensory stimulation has a negative impact on the course of dementia, the development of challenging behaviors, the shaping of the relationship work with their environment, and, above all, on the physical, cognitive, and emotional state [7].

There is ample empirical evidence that individuals with dementia in institutional care settings experience long periods of boredom, inactivity, and associated periods of loneliness [8–12]. Other observational studies document the lack of activity and inclusiveness of individuals with dementia. Accordingly, in 40 to 80 percent of the observed time periods, individuals with dementia were found sitting alone, passive, resting, dozing, sleeping, inactive, or sitting or lying in front of the television [10, 13–15]. Individuals with dementia (mis)sleep an average of five hours per day [16]. It should be noted that activation opportunities in residential care settings are reduced [17]. In an observational study, de Boer et al. examined different settings in institutional long-term care [18]. Although they found significantly more activity and social interaction in specialized institutions, even in these settings individuals with dementia appeared to spend a relatively large amount of time in passive, non-purposeful activity or inactive as explored in a survey of with proxies of individuals with dementia.

The evidence highlighted above shows that individuals with dementia experience large periods of time in their daily routine when they are left to their own. However, there is a lack knowledge on how individuals with dementia experience times without care and encounters or times in which nearly nothing obviously happens to or with them. Such knowledge would be highly relevant for health professionals and individuals' relatives to develop adequate strategies for dealing with these periods of time.

Our primary objective is to search and map the topical evidence on how individuals with dementia experience times without care and encounters; explicitly guided by the following research questions:

- What happens in times without care and encounters of individuals with dementia?
- How do individuals with dementia experience (their) times without care and encounters?

For peer review only - http://bmjopen.bmj.com/site/about/guidelines.xhtml

- How do individuals with dementia act in (their) times without care and encounters?
- How do individuals with dementia spend (their) times without care and encounters?
- How do formal and informal caregivers experience times without care and encounters of individuals with dementia?

Our secondary objective with this review is to provide evidence for the conduct of an empirically ethnography that aims to reconstruct periods of time without care and encounters (referred to as free time, in German *Freizeit*) from the perspective of the individuals with dementia and from the point of view of formal or informal carers in the institutional and domestic long-term care setting in Switzerland and Germany [19–21]. Evidence that is collected by our review may support and guide the data collection, synthesis, and interpretation within the *FreiZeit* study (separate protocol in preparation).

METHODS AND ANALYSIS

Design

We generate a living and adaptive evidence map, based on a comprehensive systematic literature search [22]. An evidence map aims at mapping out, categorizing, and charting existing topical literature without its formal critical or quality assessment or synthesis [23]. A living approach enables the continuous monitoring, consideration, and integration of newly published studies into the body of evidence throughout the duration of the *FreiZeit* project [19–21]. By an adaptive approach, we aim at enabling to modify the search strategy based on newly retrieved eligible studies or research questions that may be complemented. A detailed methodological outline is provided below, structured following the "Preferred Reporting Items for Systematic Reviews and Meta-Analyses" (PRISMA) guidance [24]. This review protocol is registered on Open Science Framework (OSF; [25]).

Eligibility criteria

We include studies on times without care and encounters of individuals with dementia from the perspective of individuals with dementia themselves and formal or informal caregivers of any design (i.e., primary studies and evidence syntheses) that were conducted in the institutional and domestic long-term care setting and that were published as journal articles in English, French, or German language without any restriction of the publication year (Table 1).

Information sources

We search MEDLINE via PubMed, CINAHL, PsycInfo via Ovid, and the Web of Science Core Collection [26]. In addition, we conduct web searches using Google Scholar and citation-based searches using Scopus (i.e., direct backward and forward citation tracking of eligible publications until no further eligible publications can be identified) [27].

Search strategy

The search strategy is based on the concepts dementia (population), times without care and encounters (phenomen of interest), and the study setting (institutional and domestic long-term care setting; Table 2)).

The collection of search terms (Table 2) is based on an initial search and meta-data of its results (i.e., title, abstract, author keyword(s), controlled vocabulary) [28], brainstorming for initial search terms with members of the *FreiZeit* study team (LA and CM), reviews that were previously worked on by members of the review team and the experience in designing dementia-specific search strings of the

For peer review only - http://bmjopen.bmj.com/site/about/guidelines.xhtml

review team [29–34], and the results of ongoing preliminary searches (meta-data, i.e., title, abstract, author keyword(s), controlled vocabulary). The controlled vocabulary search is conducted within the specific database controlled vocabulary catalogues and is supported by the use of the Yale MeSH Analyzer [35], PubMed PubReminer [36], and Coremine Medical [37] based on references retrieved by the initial and preliminary searches [28].

A PubMed search of MEDLINE is translated to other databases using the Polyglot Search tool [38] and manual confirmation or adaptions by one reviewer (JH). The database-specific search strategies (in their current form when submitting this protocol) are provided in the appendix.

For web searching via Google Scholar, we use a phrase derived from the research questions and title of our review (i.e., "dementia times without care and encounters", considering the first 20 hits). For citation tracking, we backward (search references) and forward (cited by) search all eligible studies as seed references via Scopus [39], Local Citation Network [40], and ResearchRabbit [41].

The search is carried out approximately every six months for the duration of the *FreiZeit* project (three years from 2023-2025).

Study selection and data extraction

Two reviewers (JH and TB) screen titles, abstracts, and, if necessary, full texts for eligibility using the Rayyan web app [42]. One reviewer (JH) extracts the following data based on the electronic full text article version(s): author(s) and publication year, country of conduct, study aim(s), study design, population, sample size, setting, data collection method(s), data analysis method(s), main results. Data extraction is confirmed by a second reviewer (TB), if necessary. The list of variables that are extracted is not exhaustive and may be expanded, if deemed necessary by the review and/or the *FreiZeit* team.

Critical appraisal

As this is an evidence map, we do not critically appraise the included studies.

Data charting

Key characteristics and results of the included studies are charted in a tabular format with columns that are based on the extracted variables (see chapter study selection and data extraction).

Data management

One reviewer (JH) is responsible for data and literature management. A central literature database contains all search hits. Newly identified search results, irrespective of data source, are de-duplicated against the existing literature in the database using the Citavi deduplication function [43].

ETHICS AND DISSEMINATION

For each update of the evidence, we provide a tabular overview of the studies on OSF [25]. The final version of the evidence will be aimed to publish in an open access international peer-reviewed journal. In addition, we aim at disseminating our findings in scientific and non-scientific journal articles and conference proceedings as well as formats directed to the public and decision-makers in health care.

For peer review only
For peer review only - http://bmjopen.bmj.com/site/about/guidelines.xhtml

TABLES

Table 1. Eligibility criteria following the population-interest-context (PICo) format

PICo	Inclusion criteria	Exclusion criteria
**P**opulation	Individuals with dementia<br>Formal caregivers<br>Informal caregivers	Any other
Phenomenon of **I**nterest	Times without care and encounters	Other times
**C**ontext	Institutional long-term care (e.g., nursing home, care home, assisted living, day care)<br>Domestic long-term care (i.e., home care)	Acute care<br>Primary care
Design	Any type of primary study or evidence synthesis	-
Publication type	Journal article	Any other
Publication year	Any	-
Language	English<br>French<br>German	Any other

For peer review only

For peer review only - http://bmjopen.bmj.com/site/about/guidelines.xhtml

Table 2. Collection of search terms (as of when submitting this manuscript; without any search techniques added (e.g., wildcards), without indicating controlled vocabulary)

Search component	Search terms
Dementia	ALZHEIMERS DISEASE
	DEMENTIA
Times without care and encounters	ASSESSMENT TOOL FOR OCCUPATION AND SOCIAL ENGAGEMENT
	ATOSE
	BORED
	BOREDOM
	BORING
	CARE TIME
	DCM
	DEMENTIA CARE MAPPING
	DEMENTIA-CARE MAPPING
	DISCONNECTEDNESS
	CONNECTEDNESS
	CONTACTLESS
	DETACHED
	EVERYDAY LIFE
	EXPERIENCE OF TIME
	FEELING OF TIME
	FREE TIME
	INACTIVE
	INACTIVITY
	LEISURE
	LIFEWORLD
	LONESOME
	MAASTRICHT ELECTRONIC DAILY LIFE OBSERVATION TOOL
	MEDLO-TOOL
	MENORAH PARK ENGAGEMENT SCALE
	MPES
	MONOTONY
	NO CARE
	NO CONTACT
	NO SOCIAL INTERACTION
	NOT ENGAGED
	NOT INVOLVED
	RESPITE
	RESTRAINED
	RETREATED
	SENSE OF TIME
	SOCIABILITY
	TEMPORAL PERCEPTION
	TIME PERCEPTION
	UNENGAGED
	UNOCCUPIED
	UNSTRUCTURED
	WITHDRAWAL
	WITHDRAWN
Setting (institutional and domestic long-term care)	*[institutional long-term care]*
	ASSISTED LIVING FACILITY
	CARE CENTRE
	CARE CENTER
	CARE HOME
	DAY CARE
	DAY-CARE
	DAY HOSPITAL
	DAY-HOSPITAL
	HOME FOR AGED
	HOME FOR THE AGED

For peer review only
For peer review only - http://bmjopen.bmj.com/site/about/guidelines.xhtml

	HOUSING FOR THE ELDERLY
	LONG TERM CARE
	LONGTERM CARE
	LONG-TERM CARE
	LONG-TERM RESIDENT
	LONG-TERM RESIDENTIAL
	LTC
	NURSING HOME
	OLD AGE HOME
	OLD PEOPLE'S HOME
	RESIDENT
	RESIDENTIAL AGED CARE FACILITY
	RESIDENTIAL FACILITY
	RESIDENTIAL SETTING
	REST HOME
	RETIREMENT HOME
	SENIOR-CITIZENS HOME
	SENIOR CITIZENS HOME
	[domestic long-term care]
	AMBULATORY CARE
	COMMUNITY-DWELLING
	DOMESTIC
	DOMICILIARY CARE
	HOME
	HOME BASED
	HOME CARE
	HOME HEALTH
	HOME HEALTH CARE
	HOME HEALTH NURSING
	HOME NURSING
	HOME-BASED
	HOME-CARE
	LIVING IN THE COMMUNITY
	NON-INSTITUTIONAL CARE
	NON-INSTITUTIONALIZED CARE

For peer review only

For peer review only - http://bmjopen.bmj.com/site/about/guidelines.xhtml

DECLARATIONS

Funding

Swiss National Science Foundation (Weave/Lead Agency; project number: 200919). The funder had no influence on the design, conduct, and reporting of this review.

Patient and Public Involvement

We did not involve patients or the public to design this review.

Competing interests

None to declare.

Ethics approval and consent to participate

Not applicable.

Consent for publication

Not applicable.

Availability of data and material

All data used for the design of this review is published alongside.

Authors' contributions

Substantial contributions to the conception or design of the work: All authors
Drafting the work: JH
Revising it critically for important intellectual content: All authors
Final approval of the version to be published: All authors
Agreement to be accountable for all aspects of the work in ensuring that questions related to the accuracy and integrity of any part of the work are appropriately investigated and resolved: All authors

For peer review only

REFERENCES

1. Seifert A, Schelling HR. Demenzbarometer 2018. Wissen, Einstellungen und Erfahrungen der Schweizer Bevölkerung: Ein Bericht im Auftrag des Bundesamtes für Gesundheit (BAG). 2019. https://www.zora.uzh.ch/id/eprint/170355/. Accessed 21 Apr 2023.
2. Lara E, Caballero FF, Rico-Uribe LA, Olaya B, Haro JM, Ayuso-Mateos JL, Miret M. Are loneliness and social isolation associated with cognitive decline? Int J Geriatr Psychiatry. 2019;34:1613–22. doi:10.1002/gps.5174.
3. Schockenhoff E, Wetzstein V. Relationale Anthropologie - Ethische Herausforderungen bei der Betreuung von dementen Menschen. Zeitschrift für Gerontologie und Geriatrie. 2005;38:262–7. doi:10.1007/s00391-005-0321-y.
4. Zúñiga F, Favez L, Baumann S, Kindlimann A, Oeri A, Benkert B, et al. Personal und Pflegequalität in Pflegeinstitutionen in der Deutschschweiz und Romandie: SHURP 2018 – Schlussbericht. 2021. https://shurp.unibas.ch/wp-content/uploads/2021/05/SHURP-2018-Schlussbericht-April-21-1.pdf. Accessed 27 Apr 2023.
5. Bourret EM, Bernick LG, Cott CA, Kontos PC. The meaning of mobility for residents and staff in long-term care facilities. J Adv Nurs. 2002;37:338–45. doi:10.1046/j.1365-2648.2002.02104.x.
6. Striffler C. Territorialverhalten in einem Pflegeheim: Deutungen des Wohnerlebens von Menschen mit Demenz. In: Hitzler R, editor. Ethnographische Erkundungen: Methodische Aspekte aktueller Forschungsprojekte. Wiesbaden: Springer VS; 2015. p. 43–57.
7. Scherder EJ, Bogen T, Eggermont LH, Hamers JP, Swaab DF, Scherder EJA, et al. The more physical inactivity, the more agitation in dementia. Int Psychogeriatr. 2010;22:1203–8. doi:10.1017/S1041610210001493.
8. Cohen-Mansfield J, Dakheel-Ali M, Marx MS, Thein K, Regier NG. Which unmet needs contribute to behavior problems in persons with advanced dementia? Psychiatry Res. 2015;228:59–64. doi:10.1016/j.psychres.2015.03.043.
9. El Haj M, Jardri R, Larøi F, Antoine P. Hallucinations, loneliness, and social isolation in Alzheimer's disease. Cogn Neuropsychiatry. 2016;21:1–13. doi:10.1080/13546805.2015.1121139.
10. Harper Ice G. Daily life in a nursing home. Has it changed in 25 years? J Aging Stud. 2002;16:345–59. doi:10.1016/S0890-4065(02)00069-5.
11. Theurer K, Mortenson WB, Stone R, Suto M, Timonen V, Rozanova J. The need for a social revolution in residential care. J Aging Stud. 2015;35:201–10. doi:10.1016/j.jaging.2015.08.011.
12. Nyqvist F, Cattan M, Andersson L, Forsman AK, Gustafson Y. Social capital and loneliness among the very old living at home and in institutional settings: a comparative study. J Aging Health. 2013;25:1013–35. doi:10.1177/0898264313497508.
13. den Ouden M, Bleijlevens MHC, Meijers JMM, Zwakhalen SMG, Braun SM, Tan FES, Hamers JPH. Daily (In) Activities of Nursing Home Residents in Their Wards: An Observation Study. J Am Med Dir Assoc. 2015;16:963–8. doi:10.1016/j.jamda.2015.05.016.
14. Morgan-Brown M, Brangan J, McMahon R, Murphy B. Engagement and social interaction in dementia care settings. A call for occupational and social justice. Health Soc Care Community. 2019;27:400–8. doi:10.1111/hsc.12658.
15. Perrin T. Occupational need in severe dementia: a descriptive study. J Adv Nurs. 1997;25:934–41.
16. Wood W, Womack J, Hooper B. Dying of Boredom: An Exploratory Case Study of Time Use, Apparent Affect, and Routine Activity Situations on Two Alzheimer's Special Care Units. Am J Occup Ther. 2009;63:337–50. doi:10.5014/ajot.63.3.337.
17. Buettner LL, Fitzsimmons S. Activity calendars for older adults with dementia: what you see is not what you get. Am J Alzheimers Dis Other Demen. 2003;18:215–26. doi:10.1177/153331750301800405.

For peer review only - http://bmjopen.bmj.com/site/about/guidelines.xhtml

18. Boer B de, Hamers JP, Zwakhalen SM, Tan FE, Beerens HC, Verbeek H. Green Care Farms as Innovative Nursing Homes, Promoting Activities and Social Interaction for People With Dementia. J Am Med Dir Assoc. 2017;18:40–6. doi:10.1016/j.jamda.2016.10.013.
19. Hirt J, Beer T. Project overview: FreiZeit. 2023. https://osf.io/uxt48. Accessed 21 Apr 2023.
20. Hochschule Furtwangen. Forschungsprojekt "FreiZeit". 2023. https://projekt-freizeit.com/. Accessed 21 Apr 2023.
21. Swiss National Science Foundation. Betreuungs- und begegnungsfreie Zeiträume: Methodenplurale Erkundungen zum Erleben von Personen mit Demenz. 2022. https://data.snf.ch/grants/grant/200919. Accessed 21 Apr 2023.
22. Carmona C, Carroll C, Baxter S. The move towards living systematic reviews and living guidelines in healthcare: consideration of the possibilities and challenges for living qualitative evidence syntheses. Syst Rev. 2023;12:47. doi:10.1186/s13643-023-02218-0.
23. Grant MJ, Booth A. A typology of reviews: an analysis of 14 review types and associated methodologies. Health Info Libr J. 2009;26:91–108. doi:10.1111/j.1471-1842.2009.00848.x.
24. Page MJ, McKenzie JE, Bossuyt PM, Boutron I, Hoffmann TC, Mulrow CD, et al. The PRISMA 2020 statement: An updated guideline for reporting systematic reviews. J Clin Epidemiol. 2021;134:178–89. doi:10.1016/j.jclinepi.2021.03.001.
25. Hirt J, Beer T. Exploring experiences of times without care and encounters in dementia: a living and adaptive evidence map accompanying (FreiZeit). 2023. https://osf.io/gdyz9/.
26. Hirt J, Bergmann J, Karrer M. Overlaps of multiple database retrieval and citation tracking in dementia care research: a methodological study. J Med Libr Assoc. 2021;109:275–85. doi:10.5195/jmla.2021.1129.
27. Hirt J, Nordhausen T, Appenzeller-Herzog C, Ewald H. Using citation tracking for systematic literature searching - study protocol for a scoping review of methodological studies and a Delphi study [version 3; peer review: 2 approved]. F1000Res. 2021;9:1386. doi:10.12688/f1000research.27337.3.
28. Beer T, Hirt J, Bleses HM. Eventisierte Zwischenzeiten: Technologien im pflege- und betreuungsfreien Alltag von Personen mit Demenz. In: Pfannstiel MA, Kassel K, Rasche C, editors. Innovationen und Innovationsmanagement im Gesundheitswesen: Technologien, Produkte und Dienstleistungen voranbringen. Wiesbaden: Springer Gabler; 2020. p. 633–651. doi:10.1007/978-3-658-28643-9_33.
29. Hirt J, Ballhausen N, Hering A, Kliegel M, Beer T, Meyer G. Social Robot Interventions for People with Dementia: A Systematic Review on Effects and Quality of Reporting. J Alzheimers Dis. 2021;79:773–92. doi:10.3233/JAD-200347.
30. Hirt J, Beer T. Use and impact of virtual reality simulation in dementia care education: A scoping review. Nurse Educ Today. 2020;84:104207. doi:10.1016/j.nedt.2019.104207.
31. Hirt J, Burgstaller M, Zeller A, Beer T. Needs of people with dementia and their informal caregivers concerning assistive technologies: A scoping review. Pflege. 2019;32:295–304. doi:10.1024/1012-5302/a000682.
32. Hirt J, Langer G, Wilde F, Bauernschmidt D, Meyer G, Bieber A. Technology-based counselling in dementia (TeCoDem): study protocol of a mixed-methods systematic review with qualitative comparative analysis and meta-analysis. BMJ Open. 2021;11:e054157. doi:10.1136/bmjopen-2021-054157.
33. Hirt J, Vetsch J, Heinrich S. Facilitators and barriers to implement nurse-led physical activities for people with dementia in nursing homes: a protocol for a mixed-methods systematic review. BMJ Open. 2021;11:e056353. doi:10.1136/bmjopen-2021-056353.

For peer review only - http://bmjopen.bmj.com/site/about/guidelines.xhtml

34. Karrer M, Hirt J, Zeller A, Saxer S. What hinders and facilitates the implementation of nurse-led interventions in dementia care? A scoping review. BMC Geriatr. 2020;20:127. doi:10.1186/s12877-020-01520-z.
35. Yale University. Yale MeSH Analyzer. 2023. http://mesh.med.yale.edu/. Accessed 11 Apr 2023.
36. PubMed PubReMiner. 2023. http://hgserver2.amc.nl/cgi-bin/miner/miner2.cgi. Accessed 10 Oct 2022.
37. PubGene. Coremine Medical. 2023. https://www.coremine.com/medical/. Accessed 11 Apr 2023.
38. Clark JM, Sanders S, Carter M, Honeyman D, Cleo G, Auld Y, et al. Improving the translation of search strategies using the Polyglot Search Translator: a randomized controlled trial. J Med Libr Assoc. 2020;108:195–207. doi:10.5195/jmla.2020.834.
39. Hirt J, Nordhausen T, Appenzeller-Herzog C, Ewald H. Citation tracking for systematic literature searching: A scoping review. Res Syn Meth 2023. doi:10.1002/jrsm.1635.
40. Woelfle T. Local Citation Network. 2019. https://LocalCitationNetwork.github.io. Accessed 11 May 2023.
41. Research Rabbit. ResearchRabbit. 2023. https://www.researchrabbit.ai/. Accessed 11 May 2023.
42. Ouzzani M, Hammady H, Fedorowicz Z, Elmagarmid A. Rayyan-a web and mobile app for systematic reviews. Syst Rev. 2016;5:210. doi:10.1186/s13643-016-0384-4.
43. Swiss Academic Software. Citavi. 2023. https://www.citavi.com/en. Accessed 11 Apr 2023.

For peer review only

For peer review only - http://bmjopen.bmj.com/site/about/guidelines.xhtml

APPENDIX

MEDLINE via PubMed

Last search: May 11, 2023

#	Search entry	Hits
1	ALZHEIMER*[TIAB] OR DEMENT*[TIAB] OR "DEMENTIA"[MESH]	316,947
2	"ASSESSMENT TOOL OCCUPATION ENGAGEMENT"[TIAB:~3] OR ATOSE[TIAB] OR BORED[TIAB] OR BOREDOM[TIAB] OR BORING[TIAB] OR "CARE TIME"[TIAB] OR DCM[TIAB] OR "DEMENTIA CARE MAPPING"[TIAB] OR "DEMENTIA-CARE MAPPING"[TIAB] OR DISCONNECTEDNESS[TIAB] OR CONNECTEDNESS[TIAB] OR CONTACTLESS[TIAB] OR DETACHED[TIAB] OR "EVERYDAY LIFE"[TIAB] OR "EXPERIENCE TIME"[TIAB:~1] OR "FEELING TIME"[TIAB:~1] OR "FREE TIME"[TIAB] OR INACTIV*[TIAB] OR LEISURE[TIAB] OR LIFEWORLD[TIAB] OR LONESOME[TIAB] OR "MAASTRICHT ELECTRONIC DAILY LIFE OBSERVATION TOOL"[TIAB] OR "MEDLO*"[TIAB] OR "MENORAH PARK ENGAGEMENT SCALE"[TIAB] OR MPES[TIAB] OR MONOTONY[TIAB] OR "NO CARE"[TIAB] OR "NO CONTACT"[TIAB] OR "NO SOCIAL INTERACTION"[TIAB] OR "NOT ENGAGED"[TIAB] OR "NOT INVOLVED"[TIAB] OR RESPITE[TIAB] OR RESTRAINED[TIAB] OR RETREATED[TIAB] OR "SENSE TIME"[TIAB:~1] OR SOCIABILITY[TIAB] OR "TEMPORAL PERCEPTION"[TIAB] OR "TIME PERCEPTION"[TIAB] OR UNENGAGED[TIAB] OR UNOCCUPIED[TIAB] OR UNSTRUCTURED[TIAB] OR WITHDRAWAL[TIAB] OR WITHDRAWN[TIAB] OR "LEISURE ACTIVITIES"[MESH] OR "TIME PERCEPTION"[MESH]	873,861
3	"ASSISTED LIVING"[TIAB] OR "CARE CENTRE*"[TIAB] OR "CARE CENTER*"[TIAB] OR "CARE HOME*"[TIAB] OR "DAY CARE"[TIAB] OR "DAY-CARE"[TIAB] OR "DAY HOSPITAL"[TIAB] OR "DAY-HOSPITAL"[TIAB] OR "HOME* FOR AGED"[TIAB] OR "HOME* FOR THE AGED"[TIAB] OR "HOUSING FOR THE ELDERLY"[TIAB] OR "LONG TERM CARE"[TIAB] OR "LONGTERM CARE"[TIAB] OR "LONG-TERM CARE"[TIAB] OR LTC[TIAB] OR "NURSING HOME*"[TIAB] OR "OLD AGE HOME*"[TIAB] OR "OLD PEOPLE'S HOME*"[TIAB] OR RESIDENT*[TIAB] OR "REST HOME*"[TIAB] OR "RETIREMENT HOME*"[TIAB] OR "SENIOR-CITIZENS HOME*"[TIAB] OR "SENIOR CITIZENS HOME*"[TIAB] OR "HOMES FOR THE AGED"[MESH] OR "HOUSING FOR THE ELDERLY"[MESH] OR "NURSING HOMES"[MESH] OR "RESIDENTIAL FACILITIES"[MESH]	346,417
4	AMBULATORY*[TIAB] OR "COMMUNITY-DWELLING"[TIAB] OR "DOMESTIC*"[TIAB] OR DOMICILIARY*[TIAB] OR HOME*[TIAB] OR "LIVING IN THE COMMUNITY"[TIAB] OR "NON-INSTITUTIONAL*"[TIAB] OR "ADULT DAY CARE CENTERS"[MESH] OR "HOME CARE SERVICES"[MESH]	865,928
5	#3 OR #4	1,135,311
6	#1 AND #2 AND #5	1,831

For peer review only - http://bmjopen.bmj.com/site/about/guidelines.xhtml

CINAHL

Last search: May 11, 2023

#	Search entry	Hits
1	(TI ALZHEIMER* OR AB ALZHEIMER*) OR (TI DEMENT* OR AB DEMENT*) OR (MH DEMENTIA+)	111,658
2	(TI "ASSESSMENT TOOL FOR OCCUPATION AND SOCIAL ENGAGEMENT" OR AB "ASSESSMENT TOOL FOR OCCUPATION AND SOCIAL ENGAGEMENT") OR (TI ATOSE OR AB ATOSE) OR (TI BORED OR AB BORED) OR (TI BOREDOM OR AB BOREDOM) OR (TI BORING OR AB BORING) OR (TI "CARE TIME" OR AB "CARE TIME") OR (TI DCM OR AB DCM) OR (TI "DEMENTIA CARE MAPPING" OR AB "DEMENTIA CARE MAPPING") OR (TI "DEMENTIA-CARE MAPPING" OR "DEMENTIA-CARE MAPPING") OR (TI DISCONNECTEDNESS OR AB DISCONNECTEDNESS) OR (TI CONNECTEDNESS OR AB CONNECTEDNESS) OR (TI CONTACTLESS OR AB CONTACTLESS) OR (TI DETACHED OR AB DETACHED) OR (TI "EVERYDAY LIFE" OR AB "EVERYDAY LIFE") OR (TI (EXPERIENCE W1 TIME) OR AB (EXPERIENCE W1 TIME)) OR (TI (FEELING W1 TIME) OR AB (FEELING W1 TIME)) OR (TI "FREE TIME" OR AB "FREE TIME") OR (TI INACTIV* OR AB INACTIV*) OR (TI LEISURE OR AB LEISURE) OR (TI LIFEWORLD OR AB LIFEWORLD) OR (TI LONESOME OR AB LONESOME) OR (TI "MAASTRICHT ELECTRONIC DAILY LIFE OBSERVATION TOOL" OR AB "MAASTRICHT ELECTRONIC DAILY LIFE OBSERVATION TOOL") OR (TI MEDLO* OR AB MEDLO*) OR (TI "MENORAH PARK ENGAGEMENT SCALE" OR AB "MENORAH PARK ENGAGEMENT SCALE") OR (TI "MPES OR AB "MPES) OR (TI MONOTONY OR AB MONOTONY) OR (TI RESPITE OR AB RESPITE) OR (TI RESTRAINED OR AB RESTRAINED) OR (TI RETREATED OR AB RETREATED) OR (TI (SENSE W1 TIME) OR AB (SENSE W1 TIME)) OR (TI SOCIABILITY OR AB SOCIABILITY) OR (TI "TEMPORAL PERCEPTION" OR AB "TEMPORAL PERCEPTION") OR (TI "TIME PERCEPTION" OR AB "TIME PERCEPTION") OR (TI UNENGAGED OR AB UNENGAGED) OR (TI UNOCCUPIED OR AB UNOCCUPIED) OR (TI UNSTRUCTURED OR AB UNSTRUCTURED) OR (TI WITHDRAWAL OR AB WITHDRAWAL) OR (TI WITHDRAWN OR AB WITHDRAWN) OR MH LEISURE ACTIVITIES+	156,795
3	(TI "ASSISTED LIVING" OR AB "ASSISTED LIVING") OR (TI "CARE CENTRE*" OR AB "CARE CENTRE*") OR (TI "CARE CENTER*" OR AB "CARE CENTER*") OR (TI "CARE HOME*" OR AB "CARE HOME*") OR (TI "DAY CARE" OR AB "DAY CARE") OR (TI DAY-CARE OR AB DAY-CARE) OR (TI "DAY HOSPITAL" OR AB "DAY HOSPITAL") OR (TI DAY-HOSPITAL OR AB DAY-HOSPITAL) OR (TI "HOME* FOR AGED" OR AB "HOME* FOR AGED") OR (TI "HOME* FOR THE AGED" OR AB "HOME* FOR THE AGED") OR (TI "HOUSING FOR THE ELDERLY" OR AB "HOUSING FOR THE ELDERLY") OR (TI "LONG TERM CARE" OR AB "LONG TERM CARE") OR (TI "LONGTERM CARE" OR AB "LONGTERM CARE") OR (TI "LONG-TERM CARE" OR AB "LONG-TERM CARE") OR (TI LTC OR AB LTC) OR (TI "NURSING HOME*" OR AB "NURSING HOME*") OR (TI "OLD AGE HOME*" OR AB "OLD AGE HOME*") OR (TI "OLD PEOPLE'S HOME*" OR AB "OLD PEOPLE'S HOME*") OR (TI RESIDENT* OR AB RESIDENT*) OR (TI "REST HOME*" OR AB "REST HOME*") OR (TI "RETIREMENT HOME*" OR AB "RETIREMENT HOME*") OR (TI "SENIOR-CITIZENS HOME*" OR AB "SENIOR-CITIZENS HOME*") OR (TI "SENIOR CITIZENS HOME*" OR AB "SENIOR CITIZENS HOME*") OR MH LONG TERM CARE+ OR MH NURSING HOMES+ OR MH RESIDENTIAL CARE+	164,612
4	(TI AMBULATORY* OR AB AMBULATORY*) OR (TI "COMMUNITY-DWELLING" OR AB "COMMUNITY-DWELLING") OR (TI DOMESTIC* OR AB DOMESTIC*) OR (TI DOMICILIARY* OR AB DOMICILIARY*) OR (TI HOME* OR AB HOME*) OR (TI "LIVING W2 COMMUNITY" OR AB "LIVING W2 COMMUNITY") OR (TI NON-INSTITUTIONAL* OR AB NON-INSTITUTIONAL*) OR MH AMBULATORY CARE+ OR MH DAY CARE+ OR MH HOME HEALTH CARE+	309,297
5	#3 OR #4	422,577
6	#1 AND #2 AND #5	1,355

For peer review only - http://bmjopen.bmj.com/site/about/guidelines.xhtml

PsycInfo via Ovid

Last search: May 11, 2023

#	Search entry	Hits
1	(ALZHEIMER* OR DEMENT*).TI,AB. OR EXP DEMENTIA/ OR EXP ALZHEIMER'S DISEASE/	119,312
2	("ASSESSMENT TOOL FOR OCCUPATION AND SOCIAL ENGAGEMENT" OR ATOSE OR BORED OR BOREDOM OR BORING OR "CARE TIME" OR DCM OR "DEMENTIA CARE MAPPING" OR "DEMENTIA-CARE MAPPING" OR DISCONNECTEDNESS OR CONNECTEDNESS OR CONTACTLESS OR DETACHED OR "EVERYDAY LIFE" OR (EXPERIENCE ADJ1 TIME) OR (FEELING ADJ1 TIME) OR "FREE TIME" OR INACTIV* OR LEISURE OR LIFEWORLD OR LONESOME OR "MAASTRICHT ELECTRONIC DAILY LIFE OBSERVATION TOOL" OR MEDLO* OR "MENORAH PARK ENGAGEMENT SCALE" OR MPES OR MONOTONY OR "NO CARE" OR "NO CONTACT" OR "NO SOCIAL INTERACTION" OR "NOT ENGAGED" OR "NOT INVOLVED" OR RESPITE OR RESTRAINED OR RETREATED OR (SENSE ADJ1 TIME) OR SOCIABILITY OR "TEMPORAL PERCEPTION" OR "TIME PERCEPTION" OR UNENGAGED OR UNOCCUPIED OR UNSTRUCTURED OR WITHDRAWAL OR WITHDRAWN).TI,AB. OR EXP TIME PERCEPTION/ OR EXP SOCIAL WITHDRAWAL/ OR EXP SOCIABILITY/ OR EXP LEISURE TIME/	144,028
3	("ASSISTED LIVING" OR "CARE CENTRE*" OR "CARE CENTER*" OR "CARE HOME*" OR "DAY CARE" OR DAY-CARE OR "DAY HOSPITAL" OR DAY-HOSPITAL OR "HOME* FOR AGED" OR "HOME* FOR THE AGED" OR "HOUSING FOR THE ELDERLY" OR "LONG TERM CARE" OR "LONGTERM CARE" OR "LONG-TERM CARE" OR LTC OR "NURSING HOME*" OR "OLD AGE HOME*" OR "OLD PEOPLE'S HOME*" OR RESIDENT* OR "REST HOME*" OR "RETIREMENT HOME*" OR "SENIOR-CITIZENS HOME*" OR "SENIOR CITIZENS HOME*").TI,AB. OR EXP LONG TERM CARE/ OR EXP NURSING HOMES/	101,907
4	(AMBULATORY* OR COMMUNITY-DWELLING OR DOMESTIC* OR DOMICILIARY* OR HOME* OR "LIVING ADJ2 COMMUNITY" OR NON-INSTITUTIONAL*).TI,AB. OR EXP ADULT DAY CARE/ OR EXP DAY CARE CENTERS/ OR EXP HOME CARE/	229,609
5	#3 OR #4	302,197
6	#1 AND #2 AND #5	849

For peer review only - http://bmjopen.bmj.com/site/about/guidelines.xhtml

Web of Science Core Collection

Last search: May 11, 2023

#	Search entry	Hits
1	TS=(ALZHEIMER* OR DEMENT*)	397,961
2	TS=("ASSESSMENT TOOL OCCUPATION ENGAGEMENT"[TIAB:~3] OR ATOSE OR BORED OR BOREDOM OR BORING OR "CARE TIME" OR DCM OR "DEMENTIA CARE MAPPING" OR "DEMENTIA-CARE MAPPING" OR DISCONNECTEDNESS OR CONNECTEDNESS OR CONTACTLESS OR DETACHED OR "EVERYDAY LIFE" OR (EXPERIENCE NEAR/1 TIME) OR (FEELING NEAR/1 TIME) OR "FREE TIME" OR INACTIV* OR LEISURE OR LIFEWORLD OR LONESOME OR "MAASTRICHT ELECTRONIC DAILY LIFE OBSERVATION TOOL" OR "MEDLO*" OR "MENORAH PARK ENGAGEMENT SCALE" OR MPES OR MONOTONY OR "NO CARE" OR "NO CONTACT" OR "NO SOCIAL INTERACTION" OR "NOT ENGAGED" OR "NOT INVOLVED" OR RESPITE OR RESTRAINED OR RETREATED OR (SENSE NEAR/1 TIME) OR SOCIABILITY OR "TEMPORAL PERCEPTION" OR "TIME PERCEPTION" OR UNENGAGED OR UNOCCUPIED OR UNSTRUCTURED OR WITHDRAWAL OR WITHDRAWN)	1,010,148
3	TS=("ASSISTED LIVING" OR "CARE CENTRE*" OR "CARE CENTER*" OR "CARE HOME*" OR "DAY CARE" OR "DAY-CARE" OR "DAY HOSPITAL" OR "DAY-HOSPITAL" OR "HOME* FOR AGED" OR "HOME* FOR THE AGED" OR "HOUSING FOR THE ELDERLY" OR "LONG TERM CARE" OR "LONGTERM CARE" OR "LONG-TERM CARE" OR LTC OR "NURSING HOME*" OR "OLD AGE HOME*" OR "OLD PEOPLE'S HOME*" OR RESIDENT* OR "REST HOME*" OR "RETIREMENT HOME*" OR "SENIOR-CITIZENS HOME*" OR "SENIOR CITIZENS HOME*")	490,186
4	TS=(AMBULATORY* OR "COMMUNITY-DWELLING" OR "DOMESTIC*" OR DOMICILIARY* OR HOME* OR "LIVING IN THE COMMUNITY" OR "NON-INSTITUTIONAL*")	1,341,552
5	#3 OR #4	1,727,276
6	#1 AND #2 AND #5	2,071

For peer review only - http://bmjopen.bmj.com/site/about/guidelines.xhtml

PRISMA-P (Preferred Reporting Items for Systematic review and Meta-Analysis Protocols) 2015 checklist: recommended items to address in a systematic review protocol*

Section and topic	Item No	Checklist item	Page
**ADMINISTRATIVE INFORMATION**			
Title:			
Identification	1a	Identify the report as a protocol of a systematic review	1
Update	1b	If the protocol is for an update of a previous systematic review, identify as such	NA
Registration	2	If registered, provide the name of the registry (such as PROSPERO) and registration number	2/5
Authors:			
Contact	3a	Provide name, institutional affiliation, e-mail address of all protocol authors; provide physical mailing address of corresponding author	1
Contributions	3b	Describe contributions of protocol authors and identify the guarantor of the review	10
Amendments	4	If the protocol represents an amendment of a previously completed or published protocol, identify as such and list changes; otherwise, state plan for documenting important protocol amendments	NA
Support:			
Sources	5a	Indicate sources of financial or other support for the review	10
Sponsor	5b	Provide name for the review funder and/or sponsor	10
Role of sponsor or funder	5c	Describe roles of funder(s), sponsor(s), and/or institution(s), if any, in developing the protocol	10
**INTRODUCTION**			
Rationale	6	Describe the rationale for the review in the context of what is already known	4
Objectives	7	Provide an explicit statement of the question(s) the review will address with reference to participants, interventions, comparators, and outcomes (PICO)	4f.
**METHODS**			
Eligibility criteria	8	Specify the study characteristics (such as PICO, study design, setting, time frame) and report characteristics (such as years considered, language, publication status) to be used as criteria for eligibility for the review	5
Information sources	9	Describe all intended information sources (such as electronic databases, contact with study authors, trial registers or other grey literature sources) with planned dates of coverage	5
Search strategy	10	Present draft of search strategy to be used for at least one electronic database, including planned limits, such that it could be repeated	5f./14f.

For peer review only

Study records:			
Data management	11a	Describe the mechanism(s) that will be used to manage records and data throughout the review	6
Selection process	11b	State the process that will be used for selecting studies (such as two independent reviewers) through each phase of the review (that is, screening, eligibility and inclusion in meta-analysis)	6
Data collection process	11c	Describe planned method of extracting data from reports (such as piloting forms, done independently, in duplicate), any processes for obtaining and confirming data from investigators	6
Data items	12	List and define all variables for which data will be sought (such as PICO items, funding sources), any pre-planned data assumptions and simplifications	6
Outcomes and prioritization	13	List and define all outcomes for which data will be sought, including prioritization of main and additional outcomes, with rationale	6
Risk of bias in individual studies	14	Describe anticipated methods for assessing risk of bias of individual studies, including whether this will be done at the outcome or study level, or both; state how this information will be used in data synthesis	6
Data synthesis	15a	Describe criteria under which study data will be quantitatively synthesised	NA
	15b	If data are appropriate for quantitative synthesis, describe planned summary measures, methods of handling data and methods of combining data from studies, including any planned exploration of consistency (such as I^2, Kendall's τ)	NA
	15c	Describe any proposed additional analyses (such as sensitivity or subgroup analyses, meta-regression)	NA
	15d	If quantitative synthesis is not appropriate, describe the type of summary planned	6
Meta-bias(es)	16	Specify any planned assessment of meta-bias(es) (such as publication bias across studies, selective reporting within studies)	NA
Confidence in cumulative evidence	17	Describe how the strength of the body of evidence will be assessed (such as GRADE)	NA

*** It is strongly recommended that this checklist be read in conjunction with the PRISMA-P Explanation and Elaboration (cite when available) for important clarification on the items. Amendments to a review protocol should be tracked and dated. The copyright for PRISMA-P (including checklist) is held by the PRISMA-P Group and is distributed under a Creative Commons Attribution Licence 4.0.**

From: Shamseer L, Moher D, Clarke M, Ghersi D, Liberati A, Petticrew M, Shekelle P, Stewart L, PRISMA-P Group. Preferred reporting items for systematic review and meta-analysis protocols (PRISMA-P) 2015: elaboration and explanation. BMJ. 2015 Jan 2;349(jan02 1):g7647.

For peer review only

BMJ Open

Exploring experiences of times without care and encounters in dementia: protocol for a living and adaptive evidence map

Journal:	*BMJ Open*
Manuscript ID	bmjopen-2023-075664.R1
Article Type:	Protocol
Date Submitted by the Author:	23-Aug-2023
Complete List of Authors:	Hirt, Julian; Eastern Switzerland University of Applied Sciences, Department of Health; University of Basel, Pragmatic Evidence Lab, Research Center for Clinical Neuroimmunology and Neuroscience Basel (RC2NB)<br>Adlbrecht , Laura ; Eastern Switzerland University of Applied Sciences, Department of Health<br>Maurer , Carola ; Eastern Switzerland University of Applied Sciences, Department of Health<br>Beer, Thomas ; Eastern Switzerland University of Applied Sciences, Department of Health
**Primary Subject Heading**:	Public health
Secondary Subject Heading:	Nursing
Keywords:	Dementia, Nursing Care, Systematic Review

SCHOLARONE™
Manuscripts

Exploring experiences of times without care and encounters in dementia: protocol for a living and adaptive evidence map

Julian Hirt [1,2], Laura Adlbrecht [1], Carola Maurer [1], Thomas Beer [1] for the *FreiZeit* study team

Affiliation(s)
[1] Eastern Switzerland University of Applied Sciences, Department of Health, St.Gallen, Switzerland
[2] University Hospital Basel and University of Basel, Research Center for Clinical Neuroimmunology and Neuroscience Basel (RC2NB), Basel, Switzerland

FreiZeit study team members and their affiliations: https://projekt-freizeit.com/?page_id=70

Authors' ORCID and email
Julian Hirt: 0000-0001-6589-3936, julian.hirt@ost.ch
Laura Adlbrecht: 0000-0002-7042-7523, laura.adlbrecht@ost.ch
Carola Maurer: 0000-0002-2731-7767, carola.maurer@ost.ch
Thomas Beer: -, thomas.beer@ost.ch

Correspondence
Julian Hirt, Eastern Switzerland University of Applied Sciences, Department of Health, Rosenbergstrasse 59, St.Gallen, Switzerland, julian.hirt@ost.ch

For peer review only

ABSTRACT

Introduction

Individuals with dementia spend most of the day without care, without encounters, and usually without activity. Although this has been proven in studies, there is a knowledge gap on how individuals with dementia experience these periods of time. Such knowledge would be highly relevant for health professionals and relatives to develop adequate strategies for dealing with these periods of time. The *FreiZeit* study aims to reconstruct periods of time without care and encounters from the perspective of individuals with dementia and formal and informal carers. The specific objective of this review is to provide a continuously updated overview of the topical evidence that may be used to guide data synthesis and interpretation within the *FreiZeit* study.

Methods and analysis

We conduct a living evidence map, based on a comprehensive systematic literature search in MEDLINE/PubMed, CINAHL, PsycInfo/Ovid, and Web of Science Core Collection, citation-based searches, and web searches. We include studies on times without care and encounters of individuals with dementia from the perspective of individuals with dementia themselves and formal or informal caregivers of any observational study design that were conducted in the institutional and domestic long-term care setting and published as journal article in English, French, or German language without any restriction of the publication year. One reviewer screens titles, abstracts, and full texts and extracts data. Key characteristics and results of the included studies are charted in a tabular format. The searches will be run and continuously updated throughout the duration of the overarching *FreiZeit* study (every six months for two years from 2023 to 2025).

Ethics and dissemination

Ethics approval is not required for this evidence map. We disseminate our findings via journal articles and conference proceedings as well as other formats.

Registration details

This review protocol is uploaded on Open Science Framework (OSF; DOI 10.17605/OSF.IO/GDYZ9).

Keywords

Dementia [MeSH]; Nursing Homes [MeSH]; Home Nursing [MeSH]; Home Care Services [MeSH]; Review [MeSH]

For peer review only - http://bmjopen.bmj.com/site/about/guidelines.xhtml

Strengths and limitations of this study

- A comprehensive literature search combining electronic key database searching, citation-based searching, and web searching with no restrictions on publication date and outcomes is a methodological strength of our review.
- A living approach enables the continuous updating and monitoring of newly published studies.
- By an adaptive approach, we aim at enabling to modify the search strategy based on newly retrieved eligible studies or research questions that may be complemented.

For peer review only

For peer review only - http://bmjopen.bmj.com/site/about/guidelines.xhtml

INTRODUCTION

For several years, the topic of dementia has gained an enormous position in the political, social, and scientific debate. As the Swiss Dementia Barometer 2018 shows, the majority of the population feels well informed about dementia and believes that there are good options for supporting individuals with dementia [1]. At the same time, however, there is a marginalization of dementia at various levels. A review by Lara et al. shows the connection between subjectively perceived loneliness and the risk of developing dementia [2]. Accordingly, individuals with a subjective sense of marginalization are particularly affected by dementia. They feel socially excluded even before diagnosis. On the other hand, the pathologizing or stigmatizing individuals with dementia as "dementia patients" leads to an increase in marginalization. With, despite, and because of their varying degrees of loss of cognitive and communicative abilities, they are less and less integrated into social life in the sense of social participation.

The care for individuals with dementia care is increasingly provided in a social protective environment [3], for example in a family-based, dyadic care dependency or in a care facility. In Switzerland, one out of four nursing homes cares for individuals with dementia in so-called protected living areas, which are mainly closed or technologies are used to surveil individuals who left the area [4]. As a result, individuals with dementia become location-fixated, immobile, and thus isolated [5, 6]. The lack of adequate cognitive, emotional, and sensory stimulation has a negative impact on the course of dementia, the development of challenging behaviors, the shaping of the relationship work with their environment, and, above all, on the physical, cognitive, and emotional state [7].

There is ample empirical evidence that individuals with dementia in institutional care settings experience long periods of boredom, inactivity, sleep, and associated periods of loneliness [8–11] indicating that individuals with dementia experience large periods of time in their daily routine when they are left to their own, i.e., without any encounters where they are directly addressed by another person such as caregivers, other residents, family members. However, it lacks a systematic evidence overview on the characterization, temporal and periodical extent, and how individuals with dementia and caregivers experience times without care and encounters or times in which (nearly) nothing obviously happens to and/or with individuals with dementia. Such knowledge would be highly relevant for nursing homes, health professionals, and individuals' relatives to develop adequate strategies for detecting, assessing, and dealing with these periods of time.

Our primary objective is to search and map the topical evidence on times without care and encounters of people with dementia; explicitly guided by the following research questions (RQ):

- RQ1: What happens in times without care and encounters of individuals with dementia?
- RQ2: To what temporal and periodical extent do individuals with dementia experience times without care and encounter?
- RQ3: How do individuals with dementia experience times without care and encounters from their own or foreign perspective (such as caregivers and researchers)?
- RQ4: How do formal and informal caregivers experience times without care and encounters of individuals with dementia?

Our secondary objective with this review is to provide evidence for the conduct of an empirically ethnography that aims to reconstruct periods of time without care and encounters (referred to as leisure time, in German *Freizeit*) from the perspective of the individuals with dementia and from the

For peer review only - http://bmjopen.bmj.com/site/about/guidelines.xhtml

point of view of formal or informal carers in the institutional and domestic long-term care setting in Switzerland and Germany [12–14]. Evidence that is collected by our review may support and guide the data collection, synthesis, and interpretation within the *FreiZeit* study (separate protocol in preparation; [12]).

METHODS AND ANALYSIS

Design

We generate a living and adaptive evidence map, based on a comprehensive systematic literature search [15]. An evidence map aims at mapping out, categorizing, and charting existing topical literature without its formal critical or quality assessment or synthesis [16]. A living approach enables the continuous monitoring, consideration, and integration of newly published studies into the body of evidence throughout the duration of the *FreiZeit* project [12–14]. By an adaptive approach, we aim at enabling to modify the search strategy based on newly retrieved eligible studies or research questions that may be complemented. A detailed methodological outline is provided below, structured following the "Preferred Reporting Items for Systematic Reviews and Meta-Analyses" (PRISMA) guidance [17]. This review protocol is registered on Open Science Framework (OSF; [18]).

Eligibility criteria

We include studies on times without care and encounters of individuals with dementia from the perspective of individuals with dementia themselves and formal or informal caregivers of any quantitative observational and qualitative study design (i.e., cohort study, cross-sectional study, survey, qualitative descriptive study, ethnography) that were conducted in the institutional and domestic long-term care setting and published as journal articles in English, French, or German language without any restriction of the publication year (Table 1); with no restriction to sample size and type and severity of dementia. Eligible studies refer to a sample of individuals with dementia of at least 75% of the overall study sample. A formal diagnosis of dementia is no prerequisite for individuals to be eligible for our review.

Information sources

We search MEDLINE via PubMed, CINAHL, PsycInfo via Ovid, and the Web of Science Core Collection [19]. In addition, we conduct web searches using Google Scholar and citation searching using Scopus (i.e., backward and forward citation searching of eligible publications and pertinent evidence syntheses that are retrieved during study selection until no further eligible publications can be identified) [20] and Local Citation Network (i.e., incoming and outgoing suggestions) [21].

Search strategy

The search strategy is based on the concepts dementia (population), times without care and encounters (phenomen of interest), and the study setting (institutional and domestic long-term care setting; Table 2)).

The collection of search terms (Table 2) is based on an initial search and meta-data of its results (i.e., title, abstract, author keyword(s), controlled vocabulary) [22], brainstorming for initial search terms with members of the *FreiZeit* study team (LA and CM), reviews that were previously worked on by members of the review team and the experience in designing dementia-specific search strings of the review team [23–28], and the results of ongoing preliminary searches (meta-data, i.e., title, abstract, author keyword(s), controlled vocabulary). The controlled vocabulary search is conducted within the

For peer review only - http://bmjopen.bmj.com/site/about/guidelines.xhtml

specific database controlled vocabulary catalogues and is supported by the use of the Yale MeSH Analyzer [29], PubMed PubReminer [30], and Coremine Medical [31] based on references retrieved by the initial and preliminary searches [22].

A PubMed search of MEDLINE is translated to other databases using the Polyglot Search tool [32] and manual confirmation or adaptions by one reviewer (JH). The database-specific search strategies (for the initial search in May 2023) are provided in the appendix (any updates on the database-specific search strategies will be reported on OSF [18].

For web searching via Google Scholar, we use a phrase derived from the research questions and title of our review (i.e., "dementia times without care and encounters", considering the first 20 hits). For citation searching, we backward citation search (reference list checking) and forward citation search (cited by search) all eligible studies and pertinent evidence syntheses (that are retrieved during study selection) as seed references via Scopus [33]; we perform iterative layers until no more additional relevant reference retrieves. In addition, we use the function of incoming and outgoing suggestions of the Local Citation Network to retrieve references that were not yet identified via other sources [21]. All searches are carried out approximately every six months for the duration of the *FreiZeit* project (each May and November of the years 2023 and 2024).

Study selection and data extraction

One reviewer (JH) screens titles, abstracts, and full texts for eligibility using the Rayyan web app [34] (title/abstract level by screening all references manually, i.e., without the use of integrated machine learning features) and Covidence [35] (full text level). Study selection is confirmed by a second reviewer (TB), if necessary.

One reviewer (JH) extracts the following data based on the electronic full text article version(s) using Covidence [35]: author(s) and publication year, country of conduct, study aim or research question (as expressed by authors) and which RQs are addressed, study design, population (type of participants, sample size, and type and severity of dementia (as per eligibility criteria), setting (type as expressed by authors), number of study sites, data collection (methods (e.g., observation, survey), assessment instrument, perspective, and, for observations, period of time (e.g., x days between month y and z), time frame (e.g., between x and y o'clock), and schedule (e.g., any other details provided)), data analysis methods, unit of observation (i.e., observational assessment or individuals with dementia), and main results. If the reported results of individual studies are not adequately usable for the purpose of our evidence map (i.e., depending on the way the data is presented), we track this accordingly, but we do not request data from study authors. Data extraction is confirmed by a second reviewer (TB), if necessary. The list of variables that are extracted is not exhaustive and may be expanded, if deemed necessary by the review and/or the *FreiZeit* team.

Critical appraisal

As this is an evidence map that aims at providing an overview of the available topical evidence without any quality assessment, we do not critically appraise the included studies.

Data charting

Key characteristics and results of the included studies are charted in a tabular format with columns that are based on the extracted variables (see chapter study selection and data extraction).

Data management

For peer review only - http://bmjopen.bmj.com/site/about/guidelines.xhtml

One reviewer (JH) is responsible for data and literature management. A central Citavi literature database contains all search hits [36]. Newly identified search results, irrespective of data source, are de-duplicated against the existing literature in the database using the Citavi deduplication function [36].

ETHICS AND DISSEMINATION

For each update of the evidence, we provide the applied methods and a tabular overview of the studies and their results on OSF [18]. The final version of the evidence will be aimed to publish in an open access international peer-reviewed journal.
In addition, we aim at disseminating our findings in scientific and non-scientific journal articles and conference proceedings as well as formats directed to the public and decision-makers in health care.

For peer review only

For peer review only - http://bmjopen.bmj.com/site/about/guidelines.xhtml

TABLES

Table 1. Eligibility criteria following the population-interest-context (PICo) format

PICo	Inclusion criteria	Exclusion criteria
**P**opulation	Individuals with dementia *<br>Formal caregivers **<br>Informal caregivers ***	Any other
Phenomenon of **I**nterest	Times without care and encounters	Other times
**C**ontext	Institutional long-term care (e.g., nursing home, care home, assisted living, day care)<br>Domestic long-term care (i.e., home care)	Acute care<br>Primary care
Design	Any type of quantitative observational or qualitative study	Evidence syntheses<br>Experimental/interventional research
Publication type	Journal article ****	Any other
Publication year	Any	-
Language	English<br>French<br>German	Any other

* As referred to by study authors that may mean that dementia is formally diagnosed by a physician and or assessed by health professionals such as nurses or psychologists.
** Professional caregivers such as nurses, nurse aids, or activity staff.
*** Non-paid caregivers such as family members, relatives, or friends.
**** Irrespective of whether an article was peer reviewed or not.

For peer review only

For peer review only - http://bmjopen.bmj.com/site/about/guidelines.xhtml

Table 2. Collection of search terms (as of when submitting this manuscript; without any search techniques added (e.g., wildcards), without indicating controlled vocabulary)

Search component	Search terms
Dementia	ALZHEIMERS DISEASE
	DEMENTIA
Times without care and encounters	ASSESSMENT TOOL FOR OCCUPATION AND SOCIAL ENGAGEMENT
	ATOSE
	BORED
	BOREDOM
	BORING
	CARE TIME
	DCM
	DEMENTIA CARE MAPPING
	DEMENTIA-CARE MAPPING
	DISCONNECTEDNESS
	CONNECTEDNESS
	CONTACTLESS
	DETACHED
	EVERYDAY LIFE
	EXPERIENCE OF TIME
	FEELING OF TIME
	FREE TIME
	INACTIVE
	INACTIVITY
	LEISURE
	LIFEWORLD
	LONESOME
	MAASTRICHT ELECTRONIC DAILY LIFE OBSERVATION TOOL
	MEDLO-TOOL
	MENORAH PARK ENGAGEMENT SCALE
	MPES
	MONOTONY
	NO CARE
	NO CONTACT
	NO SOCIAL INTERACTION
	NOT ENGAGED
	NOT INVOLVED
	RESPITE
	RESTRAINED
	RETREATED
	SENSE OF TIME
	SOCIABILITY
	TEMPORAL PERCEPTION
	TIME PERCEPTION
	UNENGAGED
	UNOCCUPIED
	UNSTRUCTURED
	WITHDRAWAL
	WITHDRAWN
Setting (institutional and domestic long-term care)	*[institutional long-term care]*
	ASSISTED LIVING FACILITY
	CARE CENTRE
	CARE CENTER
	CARE HOME
	DAY CARE
	DAY-CARE
	DAY HOSPITAL
	DAY-HOSPITAL
	HOME FOR AGED
	HOME FOR THE AGED

For peer review only - http://bmjopen.bmj.com/site/about/guidelines.xhtml

	HOUSING FOR THE ELDERLY
	LONG TERM CARE
	LONGTERM CARE
	LONG-TERM CARE
	LONG-TERM RESIDENT
	LONG-TERM RESIDENTIAL
	LTC
	NURSING HOME
	OLD AGE HOME
	OLD PEOPLE'S HOME
	RESIDENT
	RESIDENTIAL AGED CARE FACILITY
	RESIDENTIAL FACILITY
	RESIDENTIAL SETTING
	REST HOME
	RETIREMENT HOME
	SENIOR-CITIZENS HOME
	SENIOR CITIZENS HOME
	[domestic long-term care]
	AMBULATORY CARE
	COMMUNITY-DWELLING
	DOMESTIC
	DOMICILIARY CARE
	HOME
	HOME BASED
	HOME CARE
	HOME HEALTH
	HOME HEALTH CARE
	HOME HEALTH NURSING
	HOME NURSING
	HOME-BASED
	HOME-CARE
	LIVING IN THE COMMUNITY
	NON-INSTITUTIONAL CARE
	NON-INSTITUTIONALIZED CARE

For peer review only

For peer review only - http://bmjopen.bmj.com/site/about/guidelines.xhtml

DECLARATIONS

Funding

Swiss National Science Foundation (Weave/Lead Agency; project number: 200919). The funder had no influence on the design, conduct, and reporting of this review.

Patient and Public Involvement

We did not involve patients or the public to design this review.

Competing interests

None to declare.

Ethics approval and consent to participate

Not applicable.

Consent for publication

Not applicable.

Availability of data and material

All data used for the design of this review is published alongside.

Authors' contributions

Julian Hirt (JH) and Thomas Beer (TB) had the idea for this review and made substantial contributions to its conception. JH, Laura Adlbrecht (LA), Carola Maurer (CM), and TB designed the methodological outline for this review. JH drafted the manuscript. LA, CM, and TB critically revised it for important intellectual content. JH, LA, CM, and TB (all authors) finally approved the latest version to be published and agreed to be accountable for all aspects of the work in ensuring that questions related to the accuracy and integrity of any part of the work are appropriately investigated and resolved.

For peer review only

REFERENCES

1. Seifert A, Schelling HR. Demenzbarometer 2018. Wissen, Einstellungen und Erfahrungen der Schweizer Bevölkerung: Ein Bericht im Auftrag des Bundesamtes für Gesundheit (BAG). 2019. https://www.zora.uzh.ch/id/eprint/170355/. Accessed 21 Apr 2023.
2. Lara E, Caballero FF, Rico-Uribe LA, Olaya B, Haro JM, Ayuso-Mateos JL, Miret M. Are loneliness and social isolation associated with cognitive decline? Int J Geriatr Psychiatry. 2019;34:1613–22. doi:10.1002/gps.5174.
3. Schockenhoff E, Wetzstein V. Relationale Anthropologie - Ethische Herausforderungen bei der Betreuung von dementen Menschen. Zeitschrift für Gerontologie und Geriatrie. 2005;38:262–7. doi:10.1007/s00391-005-0321-y.
4. Zúñiga F, Favez L, Baumann S, Kindlimann A, Oeri A, Benkert B, et al. Personal und Pflegequalität in Pflegeinstitutionen in der Deutschschweiz und Romandie: SHURP 2018 – Schlussbericht. 2021. https://shurp.unibas.ch/wp-content/uploads/2021/05/SHURP-2018-Schlussbericht-April-21-1.pdf. Accessed 27 Apr 2023.
5. Bourret EM, Bernick LG, Cott CA, Kontos PC. The meaning of mobility for residents and staff in long-term care facilities. J Adv Nurs. 2002;37:338–45. doi:10.1046/j.1365-2648.2002.02104.x.
6. Striffler C. Territorialverhalten in einem Pflegeheim: Deutungen des Wohnerlebens von Menschen mit Demenz. In: Hitzler R, editor. Ethnographische Erkundungen: Methodische Aspekte aktueller Forschungsprojekte. Wiesbaden: Springer VS; 2015. p. 43–57.
7. Scherder EJ, Bogen T, Eggermont LH, Hamers JP, Swaab DF, Scherder EJA, et al. The more physical inactivity, the more agitation in dementia. Int Psychogeriatr. 2010;22:1203–8. doi:10.1017/S1041610210001493.
8. Cohen-Mansfield J, Dakheel-Ali M, Marx MS, Thein K, Regier NG. Which unmet needs contribute to behavior problems in persons with advanced dementia? Psychiatry Res. 2015;228:59–64. doi:10.1016/j.psychres.2015.03.043.
9. El Haj M, Jardri R, Larøi F, Antoine P. Hallucinations, loneliness, and social isolation in Alzheimer's disease. Cogn Neuropsychiatry. 2016;21:1–13. doi:10.1080/13546805.2015.1121139.
10. Theurer K, Mortenson WB, Stone R, Suto M, Timonen V, Rozanova J. The need for a social revolution in residential care. J Aging Stud. 2015;35:201–10. doi:10.1016/j.jaging.2015.08.011.
11. Nyqvist F, Cattan M, Andersson L, Forsman AK, Gustafson Y. Social capital and loneliness among the very old living at home and in institutional settings: a comparative study. J Aging Health. 2013;25:1013–35. doi:10.1177/0898264313497508.
12. Hirt J, Beer T. Project overview: FreiZeit. 2023. https://osf.io/uxt48. Accessed 21 Apr 2023.
13. Hochschule Furtwangen. Forschungsprojekt "FreiZeit". 2023. https://projekt-freizeit.com/. Accessed 21 Apr 2023.
14. Swiss National Science Foundation. Betreuungs- und begegnungsfreie Zeiträume: Methodenplurale Erkundungen zum Erleben von Personen mit Demenz. 2022. https://data.snf.ch/grants/grant/200919. Accessed 21 Apr 2023.
15. Carmona C, Carroll C, Baxter S. The move towards living systematic reviews and living guidelines in healthcare: consideration of the possibilities and challenges for living qualitative evidence syntheses. Syst Rev. 2023;12:47. doi:10.1186/s13643-023-02218-0.
16. Grant MJ, Booth A. A typology of reviews: an analysis of 14 review types and associated methodologies. Health Info Libr J. 2009;26:91–108. doi:10.1111/j.1471-1842.2009.00848.x.
17. Page MJ, McKenzie JE, Bossuyt PM, Boutron I, Hoffmann TC, Mulrow CD, et al. The PRISMA 2020 statement: An updated guideline for reporting systematic reviews. J Clin Epidemiol. 2021;134:178–89. doi:10.1016/j.jclinepi.2021.03.001.

For peer review only - http://bmjopen.bmj.com/site/about/guidelines.xhtml

18. Hirt J, Beer T. Exploring experiences of times without care and encounters in dementia: a living and adaptive evidence map accompanying (FreiZeit). 2023. https://osf.io/gdyz9/.
19. Hirt J, Bergmann J, Karrer M. Overlaps of multiple database retrieval and citation tracking in dementia care research: a methodological study. J Med Libr Assoc. 2021;109:275–85. doi:10.5195/jmla.2021.1129.
20. Hirt J, Nordhausen T, Appenzeller-Herzog C, Ewald H. Using citation tracking for systematic literature searching - study protocol for a scoping review of methodological studies and a Delphi study [version 3; peer review: 2 approved]. F1000Res. 2021;9:1386. doi:10.12688/f1000research.27337.3.
21. Woelfle T. Local Citation Network. 2019. https://LocalCitationNetwork.github.io. Accessed 16 Jun 2023.
22. Beer T, Hirt J, Bleses HM. Eventisierte Zwischenzeiten: Technologien im pflege- und betreuungsfreien Alltag von Personen mit Demenz. In: Pfannstiel MA, Kassel K, Rasche C, editors. Innovationen und Innovationsmanagement im Gesundheitswesen: Technologien, Produkte und Dienstleistungen voranbringen. Wiesbaden: Springer Gabler; 2020. p. 633–651. doi:10.1007/978-3-658-28643-9_33.
23. Hirt J, Ballhausen N, Hering A, Kliegel M, Beer T, Meyer G. Social Robot Interventions for People with Dementia: A Systematic Review on Effects and Quality of Reporting. J Alzheimers Dis. 2021;79:773–92. doi:10.3233/JAD-200347.
24. Hirt J, Beer T. Use and impact of virtual reality simulation in dementia care education: A scoping review. Nurse Educ Today. 2020;84:104207. doi:10.1016/j.nedt.2019.104207.
25. Hirt J, Burgstaller M, Zeller A, Beer T. Needs of people with dementia and their informal caregivers concerning assistive technologies: A scoping review. Pflege. 2019;32:295–304. doi:10.1024/1012-5302/a000682.
26. Hirt J, Langer G, Wilde F, Bauernschmidt D, Meyer G, Bieber A. Technology-based counselling in dementia (TeCoDem): study protocol of a mixed-methods systematic review with qualitative comparative analysis and meta-analysis. BMJ Open. 2021;11:e054157. doi:10.1136/bmjopen-2021-054157.
27. Hirt J, Vetsch J, Heinrich S. Facilitators and barriers to implement nurse-led physical activities for people with dementia in nursing homes: a protocol for a mixed-methods systematic review. BMJ Open. 2021;11:e056353. doi:10.1136/bmjopen-2021-056353.
28. Karrer M, Hirt J, Zeller A, Saxer S. What hinders and facilitates the implementation of nurse-led interventions in dementia care? A scoping review. BMC Geriatr. 2020;20:127. doi:10.1186/s12877-020-01520-z.
29. Yale University. Yale MeSH Analyzer. 2023. http://mesh.med.yale.edu/. Accessed 11 Apr 2023.
30. PubMed PubReMiner. 2023. http://hgserver2.amc.nl/cgi-bin/miner/miner2.cgi. Accessed 10 Oct 2022.
31. PubGene. Coremine Medical. 2023. https://www.coremine.com/medical/. Accessed 11 Apr 2023.
32. Clark JM, Sanders S, Carter M, Honeyman D, Cleo G, Auld Y, et al. Improving the translation of search strategies using the Polyglot Search Translator: a randomized controlled trial. J Med Libr Assoc. 2020;108:195–207. doi:10.5195/jmla.2020.834.
33. Hirt J, Nordhausen T, Appenzeller-Herzog C, Ewald H. Citation tracking for systematic literature searching: A scoping review. Res Syn Meth 2023. doi:10.1002/jrsm.1635.
34. Ouzzani M, Hammady H, Fedorowicz Z, Elmagarmid A. Rayyan-a web and mobile app for systematic reviews. Syst Rev. 2016;5:210. doi:10.1186/s13643-016-0384-4.
35. Covidence. Covidence. Better systematic review management. 2023. https://www.covidence.org/. Accessed 25 May 2023.

For peer review only - http://bmjopen.bmj.com/site/about/guidelines.xhtml

36. Swiss Academic Software. Citavi. 2023. https://www.citavi.com/en. Accessed 11 Apr 2023.

For peer review only

For peer review only - http://bmjopen.bmj.com/site/about/guidelines.xhtml

APPENDIX

MEDLINE via PubMed

Last search: May 11, 2023

#	Search entry	Hits
1	ALZHEIMER*[TIAB] OR DEMENT*[TIAB] OR "DEMENTIA"[MESH]	316,947
2	"ASSESSMENT TOOL OCCUPATION ENGAGEMENT"[TIAB:~3] OR ATOSE[TIAB] OR BORED[TIAB] OR BOREDOM[TIAB] OR BORING[TIAB] OR "CARE TIME"[TIAB] OR DCM[TIAB] OR "DEMENTIA CARE MAPPING"[TIAB] OR "DEMENTIA-CARE MAPPING"[TIAB] OR DISCONNECTEDNESS[TIAB] OR CONNECTEDNESS[TIAB] OR CONTACTLESS[TIAB] OR DETACHED[TIAB] OR "EVERYDAY LIFE"[TIAB] OR "EXPERIENCE TIME"[TIAB:~1] OR "FEELING TIME"[TIAB:~1] OR "FREE TIME"[TIAB] OR INACTIV*[TIAB] OR LEISURE[TIAB] OR LIFEWORLD[TIAB] OR LONESOME[TIAB] OR "MAASTRICHT ELECTRONIC DAILY LIFE OBSERVATION TOOL"[TIAB] OR "MEDLO*"[TIAB] OR "MENORAH PARK ENGAGEMENT SCALE"[TIAB] OR MPES[TIAB] OR MONOTONY[TIAB] OR "NO CARE"[TIAB] OR "NO CONTACT"[TIAB] OR "NO SOCIAL INTERACTION"[TIAB] OR "NOT ENGAGED"[TIAB] OR "NOT INVOLVED"[TIAB] OR RESPITE[TIAB] OR RESTRAINED[TIAB] OR RETREATED[TIAB] OR "SENSE TIME"[TIAB:~1] OR SOCIABILITY[TIAB] OR "TEMPORAL PERCEPTION"[TIAB] OR "TIME PERCEPTION"[TIAB] OR UNENGAGED[TIAB] OR UNOCCUPIED[TIAB] OR UNSTRUCTURED[TIAB] OR WITHDRAWAL[TIAB] OR WITHDRAWN[TIAB] OR "LEISURE ACTIVITIES"[MESH] OR "TIME PERCEPTION"[MESH]	873,861
3	"ASSISTED LIVING"[TIAB] OR "CARE CENTRE*"[TIAB] OR "CARE CENTER*"[TIAB] OR "CARE HOME*"[TIAB] OR "DAY CARE"[TIAB] OR "DAY-CARE"[TIAB] OR "DAY HOSPITAL"[TIAB] OR "DAY-HOSPITAL"[TIAB] OR "HOME* FOR AGED"[TIAB] OR "HOME* FOR THE AGED"[TIAB] OR "HOUSING FOR THE ELDERLY"[TIAB] OR "LONG TERM CARE"[TIAB] OR "LONGTERM CARE"[TIAB] OR "LONG-TERM CARE"[TIAB] OR LTC[TIAB] OR "NURSING HOME*"[TIAB] OR "OLD AGE HOME*"[TIAB] OR "OLD PEOPLE'S HOME*"[TIAB] OR RESIDENT*[TIAB] OR "REST HOME*"[TIAB] OR "RETIREMENT HOME*"[TIAB] OR "SENIOR-CITIZENS HOME*"[TIAB] OR "SENIOR CITIZENS HOME*"[TIAB] OR "HOMES FOR THE AGED"[MESH] OR "HOUSING FOR THE ELDERLY"[MESH] OR "NURSING HOMES"[MESH] OR "RESIDENTIAL FACILITIES"[MESH]	346,417
4	AMBULATORY*[TIAB] OR "COMMUNITY-DWELLING"[TIAB] OR "DOMESTIC*"[TIAB] OR DOMICILIARY*[TIAB] OR HOME*[TIAB] OR "LIVING IN THE COMMUNITY"[TIAB] OR "NON-INSTITUTIONAL*"[TIAB] OR "ADULT DAY CARE CENTERS"[MESH] OR "HOME CARE SERVICES"[MESH]	865,928
5	#3 OR #4	1,135,311
6	#1 AND #2 AND #5	1,831

For peer review only - http://bmjopen.bmj.com/site/about/guidelines.xhtml

CINAHL

Last search: May 11, 2023

#	Search entry	Hits
1	(TI ALZHEIMER* OR AB ALZHEIMER*) OR (TI DEMENT* OR AB DEMENT*) OR (MH DEMENTIA+)	111,658
2	(TI "ASSESSMENT TOOL FOR OCCUPATION AND SOCIAL ENGAGEMENT" OR AB "ASSESSMENT TOOL FOR OCCUPATION AND SOCIAL ENGAGEMENT") OR (TI ATOSE OR AB ATOSE) OR (TI BORED OR AB BORED) OR (TI BOREDOM OR AB BOREDOM) OR (TI BORING OR AB BORING) OR (TI "CARE TIME" OR AB "CARE TIME") OR (TI DCM OR AB DCM) OR (TI "DEMENTIA CARE MAPPING" OR AB "DEMENTIA CARE MAPPING") OR (TI "DEMENTIA-CARE MAPPING" OR "DEMENTIA-CARE MAPPING") OR (TI DISCONNECTEDNESS OR AB DISCONNECTEDNESS) OR (TI CONNECTEDNESS OR AB CONNECTEDNESS) OR (TI CONTACTLESS OR AB CONTACTLESS) OR (TI DETACHED OR AB DETACHED) OR (TI "EVERYDAY LIFE" OR AB "EVERYDAY LIFE") OR (TI (EXPERIENCE W1 TIME) OR AB (EXPERIENCE W1 TIME)) OR (TI (FEELING W1 TIME) OR AB (FEELING W1 TIME)) OR (TI "FREE TIME" OR AB "FREE TIME") OR (TI INACTIV* OR AB INACTIV*) OR (TI LEISURE OR AB LEISURE) OR (TI LIFEWORLD OR AB LIFEWORLD) OR (TI LONESOME OR AB LONESOME) OR (TI "MAASTRICHT ELECTRONIC DAILY LIFE OBSERVATION TOOL" OR AB "MAASTRICHT ELECTRONIC DAILY LIFE OBSERVATION TOOL") OR (TI MEDLO* OR AB MEDLO*) OR (TI "MENORAH PARK ENGAGEMENT SCALE" OR AB "MENORAH PARK ENGAGEMENT SCALE") OR (TI "MPES OR AB "MPES) OR (TI MONOTONY OR AB MONOTONY) OR (TI RESPITE OR AB RESPITE) OR (TI RESTRAINED OR AB RESTRAINED) OR (TI RETREATED OR AB RETREATED) OR (TI (SENSE W1 TIME) OR AB (SENSE W1 TIME)) OR (TI SOCIABILITY OR AB SOCIABILITY) OR (TI "TEMPORAL PERCEPTION" OR AB "TEMPORAL PERCEPTION") OR (TI "TIME PERCEPTION" OR AB "TIME PERCEPTION") OR (TI UNENGAGED OR AB UNENGAGED) OR (TI UNOCCUPIED OR AB UNOCCUPIED) OR (TI UNSTRUCTURED OR AB UNSTRUCTURED) OR (TI WITHDRAWAL OR AB WITHDRAWAL) OR (TI WITHDRAWN OR AB WITHDRAWN) OR MH LEISURE ACTIVITIES+	156,795
3	(TI "ASSISTED LIVING" OR AB "ASSISTED LIVING") OR (TI "CARE CENTRE*" OR AB "CARE CENTRE*") OR (TI "CARE CENTER*" OR AB "CARE CENTER*") OR (TI "CARE HOME*" OR AB "CARE HOME*") OR (TI "DAY CARE" OR AB "DAY CARE") OR (TI DAY-CARE OR AB DAY-CARE) OR (TI "DAY HOSPITAL" OR AB "DAY HOSPITAL") OR (TI DAY-HOSPITAL OR AB DAY-HOSPITAL) OR (TI "HOME* FOR AGED" OR AB "HOME* FOR AGED") OR (TI "HOME* FOR THE AGED" OR AB "HOME* FOR THE AGED") OR (TI "HOUSING FOR THE ELDERLY" OR AB "HOUSING FOR THE ELDERLY") OR (TI "LONG TERM CARE" OR AB "LONG TERM CARE") OR (TI "LONGTERM CARE" OR AB "LONGTERM CARE") OR (TI "LONG-TERM CARE" OR AB "LONG-TERM CARE") OR (TI LTC OR AB LTC) OR (TI "NURSING HOME*" OR AB "NURSING HOME*") OR (TI "OLD AGE HOME*" OR AB "OLD AGE HOME*") OR (TI "OLD PEOPLE'S HOME*" OR AB "OLD PEOPLE'S HOME*") OR (TI RESIDENT* OR AB RESIDENT*) OR (TI "REST HOME*" OR AB "REST HOME*") OR (TI "RETIREMENT HOME*" OR AB "RETIREMENT HOME*") OR (TI "SENIOR-CITIZENS HOME*" OR AB "SENIOR-CITIZENS HOME*") OR (TI "SENIOR CITIZENS HOME*" OR AB "SENIOR CITIZENS HOME*") OR MH LONG TERM CARE+ OR MH NURSING HOMES+ OR MH RESIDENTIAL CARE+	164,612
4	(TI AMBULATORY* OR AB AMBULATORY*) OR (TI "COMMUNITY-DWELLING" OR AB "COMMUNITY-DWELLING") OR (TI DOMESTIC* OR AB DOMESTIC*) OR (TI DOMICILIARY* OR AB DOMICILIARY*) OR (TI HOME* OR AB HOME*) OR (TI "LIVING W2 COMMUNITY" OR AB "LIVING W2 COMMUNITY") OR (TI NON-INSTITUTIONAL* OR AB NON-INSTITUTIONAL*) OR MH AMBULATORY CARE+ OR MH DAY CARE+ OR MH HOME HEALTH CARE+	309,297
5	#3 OR #4	422,577
6	#1 AND #2 AND #5	1,355

For peer review only

For peer review only - http://bmjopen.bmj.com/site/about/guidelines.xhtml

PsycInfo via Ovid

Last search: May 11, 2023

#	Search entry	Hits
1	(ALZHEIMER* OR DEMENT*).TI,AB. OR EXP DEMENTIA/ OR EXP ALZHEIMER'S DISEASE/	119,312
2	("ASSESSMENT TOOL FOR OCCUPATION AND SOCIAL ENGAGEMENT" OR ATOSE OR BORED OR BOREDOM OR BORING OR "CARE TIME" OR DCM OR "DEMENTIA CARE MAPPING" OR "DEMENTIA-CARE MAPPING" OR DISCONNECTEDNESS OR CONNECTEDNESS OR CONTACTLESS OR DETACHED OR "EVERYDAY LIFE" OR (EXPERIENCE ADJ1 TIME) OR (FEELING ADJ1 TIME) OR "FREE TIME" OR INACTIV* OR LEISURE OR LIFEWORLD OR LONESOME OR "MAASTRICHT ELECTRONIC DAILY LIFE OBSERVATION TOOL" OR MEDLO* OR "MENORAH PARK ENGAGEMENT SCALE" OR MPES OR MONOTONY OR "NO CARE" OR "NO CONTACT" OR "NO SOCIAL INTERACTION" OR "NOT ENGAGED" OR "NOT INVOLVED" OR RESPITE OR RESTRAINED OR RETREATED OR (SENSE ADJ1 TIME) OR SOCIABILITY OR "TEMPORAL PERCEPTION" OR "TIME PERCEPTION" OR UNENGAGED OR UNOCCUPIED OR UNSTRUCTURED OR WITHDRAWAL OR WITHDRAWN).TI,AB. OR EXP TIME PERCEPTION/ OR EXP SOCIAL WITHDRAWAL/ OR EXP SOCIABILITY/ OR EXP LEISURE TIME/	144,028
3	("ASSISTED LIVING" OR "CARE CENTRE*" OR "CARE CENTER*" OR "CARE HOME*" OR "DAY CARE" OR DAY-CARE OR "DAY HOSPITAL" OR DAY-HOSPITAL OR "HOME* FOR AGED" OR "HOME* FOR THE AGED" OR "HOUSING FOR THE ELDERLY" OR "LONG TERM CARE" OR "LONGTERM CARE" OR "LONG-TERM CARE" OR LTC OR "NURSING HOME*" OR "OLD AGE HOME*" OR "OLD PEOPLE'S HOME*" OR RESIDENT* OR "REST HOME*" OR "RETIREMENT HOME*" OR "SENIOR-CITIZENS HOME*" OR "SENIOR CITIZENS HOME*").TI,AB. OR EXP LONG TERM CARE/ OR EXP NURSING HOMES/	101,907
4	(AMBULATORY* OR COMMUNITY-DWELLING OR DOMESTIC* OR DOMICILIARY* OR HOME* OR "LIVING ADJ2 COMMUNITY" OR NON-INSTITUTIONAL*).TI,AB. OR EXP ADULT DAY CARE/ OR EXP DAY CARE CENTERS/ OR EXP HOME CARE/	229,609
5	#3 OR #4	302,197
6	#1 AND #2 AND #5	849

For peer review only - http://bmjopen.bmj.com/site/about/guidelines.xhtml

Web of Science Core Collection

Last search: May 11, 2023

#	Search entry	Hits
1	TS=(ALZHEIMER* OR DEMENT*)	397,961
2	TS=("ASSESSMENT TOOL OCCUPATION ENGAGEMENT"[TIAB:~3] OR ATOSE OR BORED OR BOREDOM OR BORING OR "CARE TIME" OR DCM OR "DEMENTIA CARE MAPPING" OR "DEMENTIA-CARE MAPPING" OR DISCONNECTEDNESS OR CONNECTEDNESS OR CONTACTLESS OR DETACHED OR "EVERYDAY LIFE" OR (EXPERIENCE NEAR/1 TIME) OR (FEELING NEAR/1 TIME) OR "FREE TIME" OR INACTIV* OR LEISURE OR LIFEWORLD OR LONESOME OR "MAASTRICHT ELECTRONIC DAILY LIFE OBSERVATION TOOL" OR "MEDLO*" OR "MENORAH PARK ENGAGEMENT SCALE" OR MPES OR MONOTONY OR "NO CARE" OR "NO CONTACT" OR "NO SOCIAL INTERACTION" OR "NOT ENGAGED" OR "NOT INVOLVED" OR RESPITE OR RESTRAINED OR RETREATED OR (SENSE NEAR/1 TIME) OR SOCIABILITY OR "TEMPORAL PERCEPTION" OR "TIME PERCEPTION" OR UNENGAGED OR UNOCCUPIED OR UNSTRUCTURED OR WITHDRAWAL OR WITHDRAWN)	1,010,148
3	TS=("ASSISTED LIVING" OR "CARE CENTRE*" OR "CARE CENTER*" OR "CARE HOME*" OR "DAY CARE" OR "DAY-CARE" OR "DAY HOSPITAL" OR "DAY-HOSPITAL" OR "HOME* FOR AGED" OR "HOME* FOR THE AGED" OR "HOUSING FOR THE ELDERLY" OR "LONG TERM CARE" OR "LONGTERM CARE" OR "LONG-TERM CARE" OR LTC OR "NURSING HOME*" OR "OLD AGE HOME*" OR "OLD PEOPLE'S HOME*" OR RESIDENT* OR "REST HOME*" OR "RETIREMENT HOME*" OR "SENIOR-CITIZENS HOME*" OR "SENIOR CITIZENS HOME*")	490,186
4	TS=(AMBULATORY* OR "COMMUNITY-DWELLING" OR "DOMESTIC*" OR DOMICILIARY* OR HOME* OR "LIVING IN THE COMMUNITY" OR "NON-INSTITUTIONAL*")	1,341,552
5	#3 OR #4	1,727,276
6	#1 AND #2 AND #5	2,071

For peer review only - http://bmjopen.bmj.com/site/about/guidelines.xhtml

PRISMA-P (Preferred Reporting Items for Systematic review and Meta-Analysis Protocols) 2015 checklist: recommended items to address in a systematic review protocol*

Section and topic	Item No	Checklist item	Page
**ADMINISTRATIVE INFORMATION**			
Title:			
Identification	1a	Identify the report as a protocol of a systematic review	1
Update	1b	If the protocol is for an update of a previous systematic review, identify as such	NA
Registration	2	If registered, provide the name of the registry (such as PROSPERO) and registration number	2/5
Authors:			
Contact	3a	Provide name, institutional affiliation, e-mail address of all protocol authors; provide physical mailing address of corresponding author	1
Contributions	3b	Describe contributions of protocol authors and identify the guarantor of the review	11
Amendments	4	If the protocol represents an amendment of a previously completed or published protocol, identify as such and list changes; otherwise, state plan for documenting important protocol amendments	NA
Support:			
Sources	5a	Indicate sources of financial or other support for the review	11
Sponsor	5b	Provide name for the review funder and/or sponsor	11
Role of sponsor or funder	5c	Describe roles of funder(s), sponsor(s), and/or institution(s), if any, in developing the protocol	11
**INTRODUCTION**			
Rationale	6	Describe the rationale for the review in the context of what is already known	4
Objectives	7	Provide an explicit statement of the question(s) the review will address with reference to participants, interventions, comparators, and outcomes (PICO)	4f.
**METHODS**			
Eligibility criteria	8	Specify the study characteristics (such as PICO, study design, setting, time frame) and report characteristics (such as years considered, language, publication status) to be used as criteria for eligibility for the review	5
Information sources	9	Describe all intended information sources (such as electronic databases, contact with study authors, trial registers or other grey literature sources) with planned dates of coverage	5
Search strategy	10	Present draft of search strategy to be used for at least one electronic database, including planned limits, such that it could be repeated	5f./15f.

For peer review only

Study records:			
Data management	11a	Describe the mechanism(s) that will be used to manage records and data throughout the review	6f.
Selection process	11b	State the process that will be used for selecting studies (such as two independent reviewers) through each phase of the review (that is, screening, eligibility and inclusion in meta-analysis)	6
Data collection process	11c	Describe planned method of extracting data from reports (such as piloting forms, done independently, in duplicate), any processes for obtaining and confirming data from investigators	6
Data items	12	List and define all variables for which data will be sought (such as PICO items, funding sources), any pre-planned data assumptions and simplifications	6
Outcomes and prioritization	13	List and define all outcomes for which data will be sought, including prioritization of main and additional outcomes, with rationale	6
Risk of bias in individual studies	14	Describe anticipated methods for assessing risk of bias of individual studies, including whether this will be done at the outcome or study level, or both; state how this information will be used in data synthesis	6
Data synthesis	15a	Describe criteria under which study data will be quantitatively synthesised	NA
	15b	If data are appropriate for quantitative synthesis, describe planned summary measures, methods of handling data and methods of combining data from studies, including any planned exploration of consistency (such as I^2, Kendall's τ)	NA
	15c	Describe any proposed additional analyses (such as sensitivity or subgroup analyses, meta-regression)	NA
	15d	If quantitative synthesis is not appropriate, describe the type of summary planned	6
Meta-bias(es)	16	Specify any planned assessment of meta-bias(es) (such as publication bias across studies, selective reporting within studies)	NA
Confidence in cumulative evidence	17	Describe how the strength of the body of evidence will be assessed (such as GRADE)	NA

*** It is strongly recommended that this checklist be read in conjunction with the PRISMA-P Explanation and Elaboration (cite when available) for important clarification on the items. Amendments to a review protocol should be tracked and dated. The copyright for PRISMA-P (including checklist) is held by the PRISMA-P Group and is distributed under a Creative Commons Attribution Licence 4.0.**

From: Shamseer L, Moher D, Clarke M, Ghersi D, Liberati A, Petticrew M, Shekelle P, Stewart L, PRISMA-P Group. Preferred reporting items for systematic review and meta-analysis protocols (PRISMA-P) 2015: elaboration and explanation. BMJ. 2015 Jan 2;349(jan02 1):g7647.

For peer review only
